# Multi‐Decadal Trends in Northern Lakes Show Contrasting Responses of Phytoplankton and Benthic Macroinvertebrates to Climate Change

**DOI:** 10.1111/gcb.70274

**Published:** 2025-06-09

**Authors:** Richard K. Johnson, Willem Goedkoop, Danny C. P. Lau

**Affiliations:** ^1^ Department of Aquatic Sciences and Assessment Swedish University of Agricultural Sciences Uppsala Sweden

**Keywords:** benthic macroinvertebrates, climate change, lakes, physicochemical, phytoplankton

## Abstract

Three decades of continuous monitoring of 110 lakes across Sweden revealed significant long‐term changes in physicochemical habitat and biological assemblages comprising multiple trophic levels related to climate. Mean annual air temperature increased for almost all lakes, with notable increases in the northern region. The environmental variables that showed the strongest temporal patterns were increasing water temperatures and decreasing nutrient (TP) and TOC concentrations for lakes in the north and increasing pH and TOC for lakes in the south. As hypothesized, phytoplankton and benthic macroinvertebrate (littoral and profundal) assemblages tracked climate changes directly (temperature, precipitation) and indirectly (changes in physicochemical habitat), but trends differed among the organism groups. The most pronounced changes in both magnitudes and rates of change (slopes) of the biological trends were found in the northernmost ecoregions. In these nutrient‐ and species‐poor ecosystems, taxon richness and diversity had contrasting patterns: phytoplankton and profundal macroinvertebrates had negative slopes while littoral macroinvertebrates had positive slopes. Total phytoplankton biovolume and littoral macroinvertebrate abundance had positive slopes. Spatiotemporal patterns of phytoplankton and littoral macroinvertebrates were largely correlated with temperature and nutrients but not profundal assemblages. For lakes in the south isolating climate‐induced effects was confounded by post‐acidification recovery, for example, all three organism groups correlated with pH but not with water temperature. Combined results from all of the study lakes indicated habitat‐specific responses of biological assemblages to long‐term changes in climate and physicochemical habitat. Climate change coupled with catchment vegetation and post‐acidification recovery pose heterogeneous impacts directly (temperature) and indirectly (physicochemical habitat) on lake assemblages. All three organism groups showed trends related to climate and therefore should be considered robust sentinels to gauge climate impacts directly and trophic‐level effects indirectly in these climate‐vulnerable ecosystems.

## Graphical abstract

Northern lakes are changing due to climate warming and landscape alterations. Three decades of time‐series data for 110 Swedish lakes showed that phytoplankton and bottom‐dwelling littoral and profundal faunal assemblages are responding to these environmental changes. Yet, the responses differed among the three organism groups. The most pronounced biological changes in both magnitudes and rates of change occurred in the northernmost ecoregions, and biological changes were related to warmer water temperatures and decreases in nutrient and total organic carbon concentrations. Responding predictably to environmental change, these three organism groups are robust sentinels to gauge climate impacts in these vulnerable ecosystems.

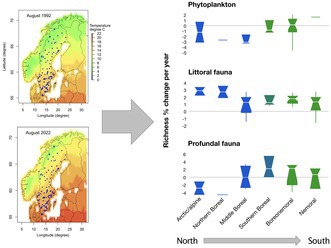

## 1 Introduction

As sentinels of environmental change, shifts in the species composition and abundance of cold‐water species are a strong indicator of lake warming (Reist et al. 2006). However, habitat conditions and biological assemblages of lakes are intricately connected with the physical, chemical, and biological processes occurring in their catchments (Magnuson et al. 2006; Williamson et al. 2008; Adrian et al. 2009), hence mechanisms underpinning change are often more complex than simple temperature‐driven physiological constraints. Climate change‐driven modifications of catchment land cover and productivity are currently altering many of these terrestrial–aquatic relationships (Elmendorf et al. 2012; Creed et al. 2018). Understanding climate impacts is therefore challenging due to the multitude, complexity, and interactions of these environmental drivers (Culp et al. 2022). For northern lakes, climate‐driven changes in catchment land cover and productivity are altering lake ecosystems (Huser et al. 2022; Nilsson et al. 2024). Declines in nutrient concentrations and increased inputs of terrestrial organic matter are two of the main drivers impacting the diversity and abundance of primary producer and consumer communities and food webs in northern lakes (Bergström and Karlsson 2019; Goedkoop et al. 2022; Lau et al. 2022; Bergström et al. 2024).

Much previous work on quantifying climate effects on lakes has relied on space‐for‐time substitution, an approach that can seriously conflate the importance of spatial and temporal drivers of variability. Long‐term datasets, on the other hand, especially if they cover broad environmental gradients, offer greater insights into the importance of regional as well as intrinsic environmental drivers on individual lakes and their biological assemblages. As high‐latitude ecosystems are expected to experience the highest rates of change in climate (IPCC 2014; Rantanen et al. 2022), long‐term monitoring datasets in northern regions are indispensable for detecting and understanding the widespread effects and consequences of climate change (Culp et al. 2022; Goedkoop et al. 2022). The Swedish national lake monitoring programme is unique as it is both spatially (covering broad latitudinal gradients from 56°N to 68°N) and temporally (> 3 decades) extensive, covering biogeographic gradients ranging from the nemoral ecoregion in the south to the arctic/alpine ecoregion in the north (Nordic Council of Ministers 1984; Gustafsson 1996). Situated in forested catchments with no or low anthropogenic impact (e.g., acidification in predominantly the southern parts of Sweden), the lakes comprise ideal model ecosystems to study climate effects on long‐term changes in biological assemblages and their physicochemical habitat.

Earlier work studying long‐term environmental changes in these lakes has shown increases in pH (Moldan et al. 2013) and TOC (Monteith et al. 2007; Eklöf et al. 2021) and decreases in nutrients (Isles et al. 2018; Huser et al. 2018) and altered stoichiometry, often attributed to changes in regional climate (temperature and precipitation) and post‐acidification recovery (e.g., Monteith et al. 2007; Eklöf et al. 2021). Focusing on single organism groups, time‐series analyses of lake phytoplankton (Bergström et al. 2020; Paltsev et al. 2024) and zooplankton (Pilotto et al. 2023; Bergström et al. 2024) have shown assemblages to be responding to these environmental changes. In this study, we quantified the biological responses (abundance and diversity metrics and community composition) of multiple organismal groups and habitats to long‐term changes in climate and lake environment, and how these responses differed spatially. We hypothesised (i) that the magnitudes and rates of change of phytoplankton correlate with declines in nutrients as well as other physicochemical drivers such as TOC and pH, (ii) that trends in benthic macroinvertebrates correlate with changes in lake productivity and physicochemical habitat, but that responses differ between littoral and profundal assemblages due to differences in habitat conditions and other potential drivers, and (iii) that the strongest patterns in both the magnitudes and rates of change of biological variables occur in the northernmost regions.

## 2 Materials and Methods

### 2.1 Study Sites

The 110 study lakes cover broad geographic gradients in latitude (range 55.5°N–68.4°N) and longitude (range 11.6°E–23.4°E), and ecosystem sizes (median lake surface area = 0.56 km^2^). Information on selected characteristics of the individual lakes is given in Table S1. On average, the lakes are characterised as circumneutral (mean pH 6.6 ± 0.69) and mesotrophic (mean TP = 14.7 ± 15.1 μg/L P and DIN 67.1 ± 70.5 μg/L N) (Table 1). Earlier work has shown the lakes to be regionally representative of the main environmental gradients occurring across the country (Johnson 1999).

**TABLE 1:** Mean (±1SD) physicochemical, climate and biological variables in the 110 northern‐LN and southern‐LN study lakes between 1992 and 2022.

Variable	Mean ± SD	Northern‐LN (*n* = 42 lakes)	Southern LN (*n* = 68 lakes)	*p*
Physicochemical				
Water temperature (°C)	12.2 ± 1.9	8.9 ± 1.8	12.3 ± 1.2	***
TP (μg/L P)	14.7 ± 15.4	8.06 ± 5.46	18.3 ± 17.6	***
DIN (μg/L N)	67.1 ± 70.5	35.3 ± 16.9	92.2 ± 85.7	***
Ca (mg/L)	5.97 ± 10.7	2.93 ± 2.33	7.93 ± 13.3	**
pH	6.6 ± 0.69	6.6 ± 0.40	6.5 ± 0.82	ns
Conductivity (mS/m)	5.95 ± 7.39	2.64 ± 1.29	8.09 ± 8.85	***
Colour (abs 420 nm)	0.132 ± 0.133	0.105 ± 0.091	0.149 ± 0.148	ns
TOC (mg/L C)	9.40 ± 4.84	6.83 ± 4.18	10.9 ± 4.55	***
Climate proxies				
Annual air temperature (°C)	4.8 ± 3.0	1.49 ± 2.01	6.98 ± 0.87	***
Annual precipitation (mm/mo)	55.8 ± 10.2	53.2 ± 6.78	57.4 ± 11.6	ns
Phytoplankton				
Richness	38.9 ± 12.4	37.3 ± 10.6	39.8 ± 13.4	ns
Biovolume (mm^3^/L)	1.48 ± 2.43	0.51 ± 0.73	2.08 ± 2.89	***
Hill diversity (N2)	17.3 ± 7.10	17.6 ± 5.71	17.2 ± 7.87	ns
Euclidean distance between years	0.360 ± 0.092	0.348 ± 0.098	0.367 ± 0.098	ns
Cyanobacteria (%)	8.16 ± 10.4	4.14 ± 4.67	10.6 ± 12.1	***
Littoral macroinvertebrates				
Richness	39.8 ± 10.2	36.4 ± 10.4	41.9 ± 9.57	*
Total abundance (NPUE)	283 ± 177	243 ± 307	307 ± 201	ns
Hill diversity (N2)	16.8 ± 4.33	15.6 ± 4.86	17.5 ± 3.84	ns
Euclidean distance between years	0.319 ± 0.089	0.348 ± 0.114	0.300 ± 0.064	ns
Profundal macroinvertebrates				
Richness	8.0 ± 4.2	9.2 ± 3.9	7.2 ± 4.2	**
Total abundance (ind./m^2^)	2438 ± 3132	1134 ± 1047	3243 ± 3683	***
Hill diversity (N2)	4.0 ± 1.70	4.7 ± 1.58	3.5 ± 1.61	***
Euclidean distance between years	0.586 ± 0.338	0.632 ± 0.298	0.557 ± 0.360	*

Abbreviation: ns = not significant.

* *p* < 0.05.

** *p* < 0.01.

*** *p* < 0.001.

### 2.2 Sampling Methods

#### 2.2.1 Water Chemistry

Mid‐lake surface water samples (0.5 m) were taken annually during four sampling intervals (March to November, representing spring, summer, autumn, and winter seasons) over a 31‐year study period between 1992 and 2022 using a Plexiglas sampler. Samples were kept cool during transport to the laboratory where they were analyzed for total phosphorus (TP, μg/L P), dissolved inorganic nitrogen (DIN, NH_4_ + NO_2_ + NO_3_ μg/L N), total organic carbon (TOC, mg/L C; of which > 90% is dissolved organic carbon in these lakes (Köhler et al. 2002)) and water colour (measured as absorbance of filtered water at 420 nm/5 cm cuvette). Water temperature (°C), electrical conductivity (mS/m) and potential of hydrogen (pH) were measured in situ. We focused on these physicochemical variables as we anticipated they would be responding to climate change and would affect biological assemblages. All physicochemical analyses were performed at the Department of Aquatic Sciences and Assessment following international (ISO) or European (EN) standards (Fölster et al. 2014). In addition, detailed information concerning methods and quality control can be found at https://www.slu.se/en/departments/aquatic‐sciences‐assessment/laboratories/vattenlabb2/.

#### 2.2.2 Phytoplankton

Phytoplankton was sampled quantitatively in late summer (August) with a tube sampler from the epilimnion in the open‐water mid‐lake area. Taxonomic identifications and enumerations were done under an inverted microscope using a modified Utermöhl technique (Olrik et al. 1998). Biovolumes (mm^3^/L) for individual species were calculated using geometric shapes following Blomqvist and Herlitz (1998) and summed to obtain total phytoplankton biovolume (BioV).

#### 2.2.3 Macroinvertebrates

Benthic macroinvertebrate samples were collected in autumn (mainly October/November) from littoral and profundal habitats (Fölster et al. 2014). For littoral habitats, macroinvertebrates were collected using standardized kick sampling with a hand net (0.5 mm mesh). Five standardized kick samples (20‐s duration, 1 m long, ca 0.5–1 m depth) were taken from stony, vegetation‐free sites in each lake. Profundal macroinvertebrate collections consisted of five Ekman grab samples (247 cm^2^) taken within a 150 m × 150 m quadrate situated over the deepest area of the lake, washed through a 0.5 mm mesh, and preserved in 70% ethanol (final concentration). In the laboratory, samples were sorted using 10× magnification, and the macroinvertebrates were counted and identified using dissecting and light microscopes. Organisms were identified generally to the species level, except for Oligochaeta and some chironomid larvae. Profundal abundances are reported as numbers per m^2^ (ind/m^2^) and littoral abundances as numbers per unit effort (NPUE). Mean values of the five replicate samples were used in our analyses.

Sampling and analyses of all abiotic and biological variables were based on standard protocols throughout the study period. These protocols are quality controlled and certified by the Swedish Board for Accreditation and Conformity Assessment (SWEDAC; https://www.swedac.se/). Each year, uniformity in sampling was streamlined by distributing instructions (e.g., sampling dates) to those involved in the monitoring programme. All physicochemical and biological analyses were performed at the Department of Aquatic Sciences and Assessment following international (ISO) or European (EN) standards (Fölster et al. 2014). Detailed information concerning methods and quality control can be found at https://www.slu.se/en/departments/aquatic‐sciences‐assessment/laboratories/vattenlabb2/.

Relationships of phytoplankton and benthic macroinvertebrate assemblages with climate and lake physicochemical conditions were analyzed using four biological variables: number of taxa, biovolume (phytoplankton BioV) or abundance (littoral and profundal macroinvertebrates), diversity (Hill's N2 calculated using Canoco) and Euclidean distance (a measure of between‐year variability in assemblage composition). Between‐year Euclidean distance (E‐dist) was calculated as the distance between two consecutive years of detrended correspondence (DC) axes 1–3 scores derived using Canoco software (version 5.12, ter Braak and Smilauer 2018; Šmilauer and Lepeš 2014) as *d* = √((*x*_2_ −*x*_1_)^2^ + (*y*_2_ − *y*_1_)^2^ + (*z*_2_ − *z*_1_)^2^), where *x*_1_, *y*_1_, *z*_1_ represent DC axis scores one, two, and three, respectively, at any year and *x*_2_, *y*_2_, *z*_2_ represent DC axis scores one, two, and three, respectively, for the previous year. For phytoplankton, we also included a fifth metric (i.e., the relative biovolume (%) of cyanobacteria), as recent studies have found this metric to be increasing in northern lakes in general (Paltsev et al. 2024) and specifically in Swedish lakes (Freeman et al. 2020), with increases related to changes in climate. In addition, relationships between phytoplankton and macroinvertebrate assemblages and climate and physicochemical variables were analyzed using constrained ordination.

Data for monthly average daily mean air temperature and precipitation were extracted from the interpolated Climate Research Unit gridded Time Series dataset (version 4.06; Harris et al. 2020; https://crudata.uea.ac.uk/cru/data/hrg/) for 0.5° latitude by 0.5° longitude grid cells corresponding to the geographic locations of the lakes. Extracted data were used to calculate annual mean air temperature (°C) and precipitation (mm per month for each lake in each year).

### 2.3 Statistical Analyses

#### 2.3.1 Time‐Series Analyses

To analyse long‐term annual trends in climate (mean annual air temperature and precipitation), physicochemical and biological variables for the period 1992–2022, we used the non‐parametric seasonal Mann–Kendall test (Hirsch et al. 1982). Monthly averages were used when several samples had been collected each month, for example, surface water physicochemical variables and phytoplankton. Temporal trends (unit/year) were estimated with the Theil‐Sen estimator (Sen 1968) and expressed as relative annual changes (%/year) calculated as Sen's slope/inter‐annual mean value × 100 individually for each lake. Only significant slopes (Kendall's tau, two‐sided test, *p* < 0.05) were used in regional comparisons (i.e., Wilcoxon and Kruskal–Wallis tests), while all slopes were used in multivariate ordinations (see below).

**FIGURE 1:**
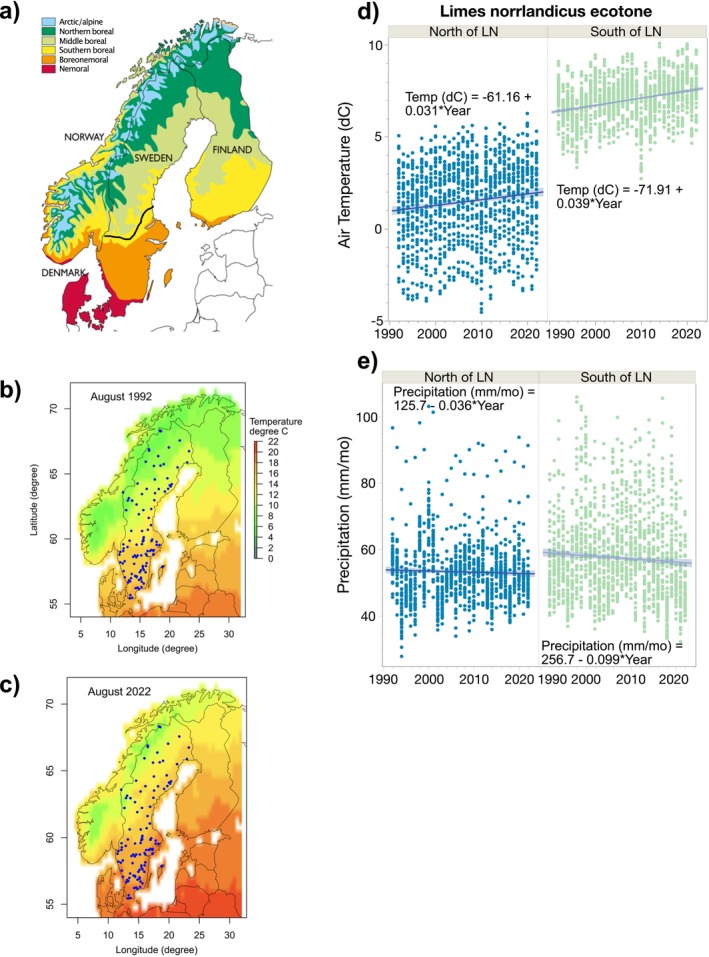
Location of the six major ecoregions of the Nordic countries with the approximate location of the Limes Norrlandius (LN) ecotone is shown in black (a), August mean annual air temperatures for 1992 (b) and 2022 (c) and mean annual air temperature (d) and precipitation (e) from 1992 to 2022 for the 110 study lakes. The panels show lakes north or south of the LN ecotone. Map lines delineate study areas and do not necessarily depict accepted national boundaries.

We analysed if trends in physicochemical and biological variables, both magnitudes (absolute values) and rates of change (%/year), were related to large‐scale spatial patterns in climate and landscape. Two spatial delineations were used to partition natural variability: the Limes Norrlandicus ecotone (e.g., Bernes 2011) and the six major ecoregions (Nordic Council of Ministers 1984; Gustafsson 1996) of Sweden (Figure 1a). The Limes Norrlandicus ecotone (LN) is a transition zone around 60°N (Bernes 2011) that mainly delineates the southern (e.g., nemoral and boreonemoral) and northern (e.g., northern boreal and arctic/alpine) ecoregions in Sweden and coincides with the approximate northernmost distribution of the oak ( *Quercus robur* ). South of the LN ecotone, winters are milder and vegetation consists predominantly of deciduous and mixed forests, while north of the LN ecotone, mean annual air temperatures are markedly lower and coniferous forests and tundra predominate. Hereafter, lakes south of the LN ecotone are referred to as southern‐LN lakes, and lakes north of the LN ecotone are referred to as northern‐LN lakes. Ecoregions of the Nordic countries, based mainly on the natural terrestrial vegetation, climate, and soil, range from the nemoral zone in the south to the arctic region in the north. The nemoral region is characterized by deciduous forests and a relatively long growth period, while the arctic/alpine complex is much colder and characterized by tundra and shrub vegetation above the tree line and stands of mountain birch ( *Betula pubescens* var. *czerepanovii*) at lower elevations. Earlier studies have shown these spatial regionalisations to partition natural variability of lake assemblages and functional guilds (Johnson and Goedkoop 2002; Johnson et al. 2004; Hallstan et al. 2013). Differences in physicochemical and biological variables in relation to the LN ecotone and among the six ecoregions were analysed with Wilcoxon and Kruskal–Wallis tests.

#### 2.3.2 Principal Components Analysis (PCA)

To visualize differences between the study lakes and reduce the dimensionality of abiotic (i.e., physicochemical and climate) variables, principal component analysis (PCA) on *Z*‐score standardized abiotic variables and correlation (Pearson Product–Moment) were used. PCA was run on abiotic and biological variables and Sen's slopes (%/year), including non‐significant slopes, to assess spatiotemporal patterns and relationships.

#### 2.3.3 Constrained Ordinations

Multivariate regressions were used to quantify the fractions of variation in phytoplankton and littoral and profundal macroinvertebrate assemblages that could be explained by three groups of explanatory variables (physicochemical, year, and climate) (ter Braak 1995). Detrended correspondence analysis showed species turnover or gradient lengths for the first three axes ranged from 2.9 to 3.4 for phytoplankton assemblages, 2.5–4.1 for littoral macroinvertebrate assemblages, and 4.9–5.4 for profundal macroinvertebrate assemblages. Accordingly, canonical correspondence analysis (CCA) and partial CCA (pCCA) were run for species assemblages. Redundancy analysis (RDA) using all slopes (i.e., including non‐significant slopes) was run to quantify variation in the 13 biological variables that could be explained by the 10 environmental variables.

Variation partitioning (pCCA) was used to quantify the temporal variability in biological assemblages that was explained uniquely by each of the three groups of abiotic variables (physicochemical, year and climate proxies), the shared variance (e.g., variance shared between each combination of two groups), and the joint variance or the variance explained by the three explanatory matrices (Peres‐Neto et al. 2006). The relative importance of each of the three groups of explanatory variables was evaluated using the adjusted *R* ^2^, providing unbiased estimates of the explained variation (Peres‐Neto et al. 2006). Down weighting of rare taxa and Monte Carlo permutation tests with 999 permutations were used to test the significance of the three variable groups and their joint variance on taxon composition, while the approach does not allow testing for significance of the shared fraction (Peres‐Neto et al. 2006).

Canonical correspondence analysis (CCA), to quantify variation in lake assemblages, and redundancy analysis (RDA), to quantify variation in the 13 biological variables, were run using forward selection to obtain the most parsimonious five‐variable model explained by eight physicochemical and two climate variables for all lakes and for northern‐LN and southern‐LN lakes separately. All ordinations were run using Hellinger‐transformed biovolume (phytoplankton) and abundance (littoral and profundal macroinvertebrates) data, down weighting of rare species, and Monte Carlo permutation tests. Multivariate regressions were done using Canoco software (version 5.12, ter Braak and Smilauer 2018; Šmilauer and Lepeš 2014).

Similarity analysis (SIMPER) was used to identify taxa that discriminated differences in assemblage composition in relation to the LN ecotone and the six ecoregions (Clarke 1993). SIMPER was run on Bray–Curtis dissimilarity matrices (Anderson 2001) based on Hellinger‐transformed data. SIMPER analyses were done using PAST software version 4.15 (Hammer et al. 2001).

Other statistical analyses were done in JMP 17.0.0 (SAS Institute Inc. JMP 2022). Climate and physicochemical data were log_10_(*x* + 1) transformed to approximate a normal distribution prior to the analyses.

## 3 Results

### 3.1 Magnitudes and Rates of Change of Physicochemical Variables

Linear regression revealed significant long‐term increases in air temperature and decreases in precipitation from 1992 to 2022 (Figure 1). Mean annual air temperature increased by 0.036°C/year, and differences in relation to the LN ecotone were significant (Wilcoxon test, *p* < 0.0001) (Figure 1b–d). North of the LN ecotone, air temperatures averaged 1.49°C ± 2.12°C, and south of the LN ecotone, 6.98°C ± 1.12°C. Mean annual precipitation decreased by −0.075 mm/year over the course of the study, and in the south, precipitation was slightly higher (57.4 ± 13.6 mm/year, mean ± SD) than the north (53.2 ± 9.35 mm/year) (Wilcoxon test, *p* < 0.0001) (Figure 1e).

Six physicochemical variables and one climate variable differed between the northern‐LN and southern‐LN lakes (Table 1). Southern‐LN lakes were on average 3°C warmer, 2.3× more nutrient‐rich, 1.4× browner, and had 3× higher conductivity than lakes in the north. Partitioning spatial variability by ecoregions revealed that differences between the northern‐LN and southern‐LN lakes were largely driven by higher magnitudes in the southern lakes (Table S2). For example, epilimnetic TP concentrations were on average 7× higher in lakes in the southernmost nemoral ecoregion than those in the northernmost arctic/alpine ecoregion (27.6 and 4.0 μg/L P, respectively), Ca concentrations were 6.3× higher (18.6 and 2.96 mg/L, respectively) and surface water temperatures were 5.6°C higher (12.4°C and 6.8°C, respectively).

On average, 40% of the lakes showed significant trends for eight physicochemical variables, and more than 50% of the lakes showed significant trends for three of these variables: conductivity, Ca (both 64%), and TOC (56%) (Table 2). The main patterns were summarised as increasing trends in variables associated with lake browning (water colour in 33% and TOC in 53% of the lakes) and pH (30%), and decreasing trends in conductivity (60%), Ca (56%) and nutrients (TP 41% and DIN 32%). Slopes of three variables (Ca and conductivity were higher in southern‐LN lakes and TOC was higher in northern‐LN lakes) differed between the northern‐LN and southern‐LN lakes (*p* < 0.05) while slopes of TP were borderline significant (*p* < 0.06).

**TABLE 2:** Number of significant trends (Mann–Kendall analysis) for physicochemical and biological variables in 110 the northern‐LN and southern‐LN study lakes between 1992 and 2022.

Variable	Positive	Negative	Total	% Slope (mean ± SD)			
				Mean ± SD	Northern‐LN	Southern‐LN	*p*
Physicochemical							
Water temperature (°C)	18	1	19	0.888 ± 0.579	1.05 ± 0.479	0.426 ± 0.635	a
TP (μg/L P)	5	45	50	−1.66 ± 1.76	−2.05 ± 1.88	−1.30 ± 1.60	a
DIN (μg/L N)	4	35	39	−2.09 ± 1.74	−2.17 ± 1.07	−1.92 ± 2.68	ns
Ca (mg/L)	9	61	70	−0.790 ± 0.835	0.021 ± 1.025	−1.09 ± 0.489	***
pH	33	4	37	0.209 ± 0.320	−0.054 ± 0.124	0.253 ± 0.344	ns
Conductivity (mS/m)	4	66	70	−0.790 ± 0.607	−0.457 ± 0.816	−0.932 ± 0.429	*
Water colour (abs 420 nm)	36	3	39	0.060 ± 0.058	0.049 ± 0.025	0.062 ± 0.062	ns
TOC (mg/L C)	58	3	61	0.996 ± 0.682	0.347 ± 0.916	1.19 ± 0.452	***
Air temperature (°C)	109	0	109	1.52 ± 8.57	1.02 ± 3.31	0.55 ± 0.074	***
Precipitation (mm/mo)	0	0	0	NA	NA	NA	NA
Phytoplankton							
Richness	13	17	30	−0.544 ± 1.87	−2.20 ± 1.55	−0.131 ± 1.73	**
Biovolume (mm^3^/L)	20	15	35	−0.387 ± 3.07	1.77 ± 1.61	−1.37 ± 3.10	**
Hill diversity (N2)	18	14	32	0.744 ± 2.74	−1.12 ± 2.96	1.72 ± 2.07	**
Euclidean distance years	12	3	15	2.13 ± 2.89	3.73 ± 0.618	0.742 ± 3.42	*
Cyanobacteria (%)	24	5	29	2.45 ± 6.02	2.71 ± 3.76	2.31 ± 7.02	ns
Littoral macroinvertebrates							
Richness	45	5	50	1.60 ± 1.40	1.49 ± 1.44	1.65 ± 1.41	ns
Abundance (NPUE)	38	8	46	3.23 ± 3.75	2.84 ± 3.93	3.42 ± 3.72	ns
Hill diversity (N)	26	9	35	0.672 ± 1.82	−0.108 ± 2.50	0.942 ± 1.48	ns
Euclidean distance among years	2	18	20	−3.31 ± 2.96	−4.35 ± 1.05	−2.87 ± 3.42	*
Profundal macroinvertebrates							
Richness	11	8	19	0.135 ± 3.03	−0.898 ± 2.90	1.07 ± 2.99	ns
Abundance (ind/m^2^)	19	12	31	0.472 ± 4.30	−1.07 ± 4.79	1.21 ± 3.95	ns
Hill diversity (N2)	12	13	25	0.052 ± 1.87	−0.321 ± 2.15	0.196 ± 1.80	ns
Euclidean distance years	9	14	23	−0.204 ± 5.72	1.36 ± 5.47	−1.21 ± 5.84	ns

*Note:* Mean % Sen's slopes (±1SD) were calculated using significant trends, and comparisons between the northern‐LN and southern‐LN lakes were made using only significant slopes. ns = not significant (*p* ≥ 0.06).

Abbreviation: NA = not applicable.

^a^ 0.05 ≤ *p* < 0.06.

* *p* < 0.05.

** *p* < 0.01.

*** *p* < 0.001.

**FIGURE 2:**
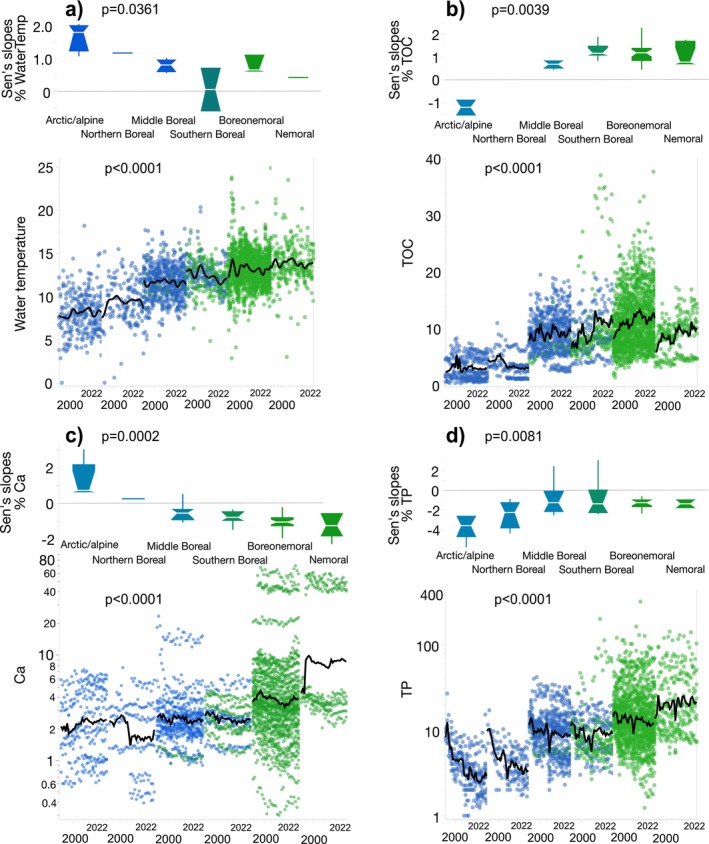
Time‐series plots and Sen's slopes (% per year) of selected physicochemical variables (a) water temperature (°C), (b) TOC (mg/L C), (c) Ca (mg/L), and (d) TP (μg/L P). Green symbols show southern‐LN and blue symbols show northern‐LN lakes. Only Sen's slopes that were significant (*p* < 0.05) are plotted. *p* values show results from a Kruskal–Wallis tests. Black lines show a 4‐year moving average.

Analysis of ecoregional patterns showed that seven of the eight physicochemical variables differed (only pH was not significant) (Table S2). Generally, magnitudes and trends in the two northernmost ecoregions differed from the other ecoregions (Figure 2). For example, across the six ecoregions, the general patterns observed were positive slopes in surface water temperatures and TOC (except for the arctic/alpine ecoregion) and negative slopes in Ca (except for the arctic/alpine ecoregion) and TP. The greatest number of significant decreases in Ca were noted in the boreonemoral ecoregion (mean slopes of −1.1% ± 0.47%/year in 35.5% of the lakes) which coincided with the highest number of positive slopes for TOC in this ecoregion (0.78% ± 0.87%/year in 31.8% of the lakes). Noteworthy were the almost consistent decreases in TP across all ecoregions, although five lakes (4.6%) had increasing trends. Arctic/alpine and northern boreal lakes had much stronger negative slopes in TP (−3.8% ± 1.2%/year and −2.5% ± 1.4%/year, respectively) than those in the other ecoregions.

### 3.2 Magnitudes and Rates of Change of Biological Variables

Seven of the 13 biological variables differed between the northern‐LN and southern‐LN lakes (Table 1), with the northern‐LN lakes having higher profundal taxon richness and diversity (1.3× for both) and slightly greater between‐year shifts in assemblage composition (E‐dist) (1.1×). Conversely, southern‐LN lakes had higher profundal macroinvertebrate abundances (2.9×), littoral macroinvertebrate taxon richness (1.2×), phytoplankton BioV (4.1×) and % cyanobacteria in phytoplankton (2.6×). Differences in biological variables in relation to the LN ecotone were largely due to lower values in arctic/alpine and northern boreal ecoregions (Table S2). For example, the ultraoligotrophic lakes in the arctic/alpine ecoregion had lower taxon richness of phytoplankton (28 ± 9) and littoral macroinvertebrates (25 ± 9) than lakes in the nemoral ecoregion (43 ± 12 and 42 ± 8, respectively). Conversely, the more nutrient‐rich lakes in the nemoral ecoregion had much higher phytoplankton BioV (37×) and % cyanobacteria (9.2×), as well as littoral (3.2×) and profundal (4.6×) macroinvertebrate abundances than arctic/alpine lakes. Compared to physicochemical variables, fewer significant trends were found for the 13 biological variables, and, except for littoral variables, no clear patterns were evident between the number of positive and negative trends of the individual variables (Table 2).

#### 3.2.1 Phytoplankton

**FIGURE 3:**
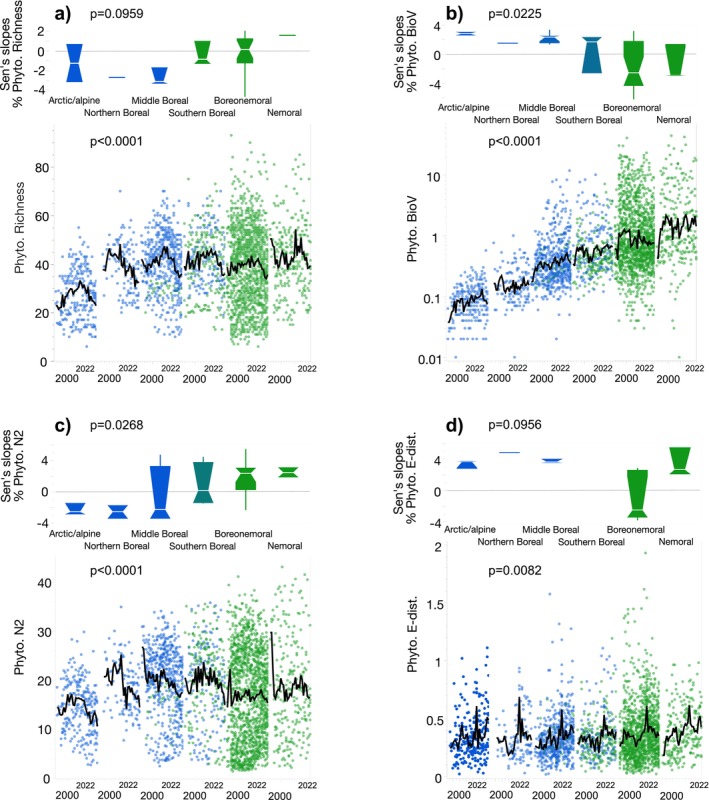
Time‐series plots and Sen's slopes (% per year) of phytoplankton (a) richness, (b) biovolume (BioV), (c) Hill's diversity (N^2^), and (d) between‐year Euclidean distance (E‐dist). Green symbols show southern‐LN and blue symbols show northern‐LN lakes. Only Sen's slopes that were significant (*p* < 0.05) are plotted. *p* values show results from a Kruskal–Wallis tests. Black lines show a 4‐year moving average.

More than 25% of the lakes had significant trends in phytoplankton taxon richness, diversity, BioV, and % cyanobacteria, and, except for % cyanobacteria, spatiotemporal patterns were significantly related to the LN ecotone (Table 2). In line with our predictions, the clearest long‐term changes were noted for the 11 northern‐LN lakes, characterised by decreasing taxon richness (5 of 6 lakes with significant trends) and diversity (9 of 11 lakes), and by increasing phytoplankton BioV (10 of 11 lakes), as well as greater between‐year shifts in assemblage composition (7 of 7 lakes). Although % cyanobacteria did not differ significantly between the northern‐LN and southern‐LN lakes, 82.8% (24 of 29 lakes) had positive slopes: 8 northern‐LN lakes and 16 southern‐LN lakes. Ecoregional differences were also observed for four of the five phytoplankton variables, as well as significant differences in slopes for phytoplankton BioV and diversity (Figure 3, for % cyanobacteria see Table S2). Phytoplankton BioV showed a gradual south‐to‐north decrease, although only positive slopes were found in the northern‐LN lakes (2.2% ± 0.68%/y for 10 lakes: arctic/alpine [*n* = 2 lakes], northern boreal [*n* = 1 lake] and middle boreal [*n* = 7 lakes] ecoregions), while, conversely, both positive and negative slopes were found for the southern‐LN lakes (Figure 3b). Significant trends in phytoplankton diversity for the northern‐LN lakes were mostly negative, while those for the southern‐LN lakes were mainly positive (Figure 3c). Negative slopes in diversity were found in all five lakes in arctic/alpine and northern boreal ecoregions (−2.4% ± 0.83%/year) while most lakes in the boreonemoral and nemoral ecoregions showed positive slopes (2.80% ± 1.07%/year in 15 of total 19 lakes with significant trends).

SIMPER results showed similarities between the top 10 ranked taxa discriminating between the northern‐LN and southern‐LN lakes and among the six ecoregions (Table S3a,b). Of the top 10 taxa, seven overlapped, with *Gonyostomum semen* (Raphidophyceae) ranked first, followed by *Pseudopediastrum* (Hydrodictyaceae) and *Gymnodinium uberrimum* (Dinophyceae): these three taxa contributed to c. 10% of the dissimilarities. *G. semen* was more abundant in the southern‐LN lakes and was absent in arctic/alpine or northern boreal lakes. The green algae *Pseudopediastrum* and several Cryptophyceae taxa had higher abundances in the two northernmost ecoregions.

#### 3.2.2 Littoral Macroinvertebrates

**FIGURE 4:**
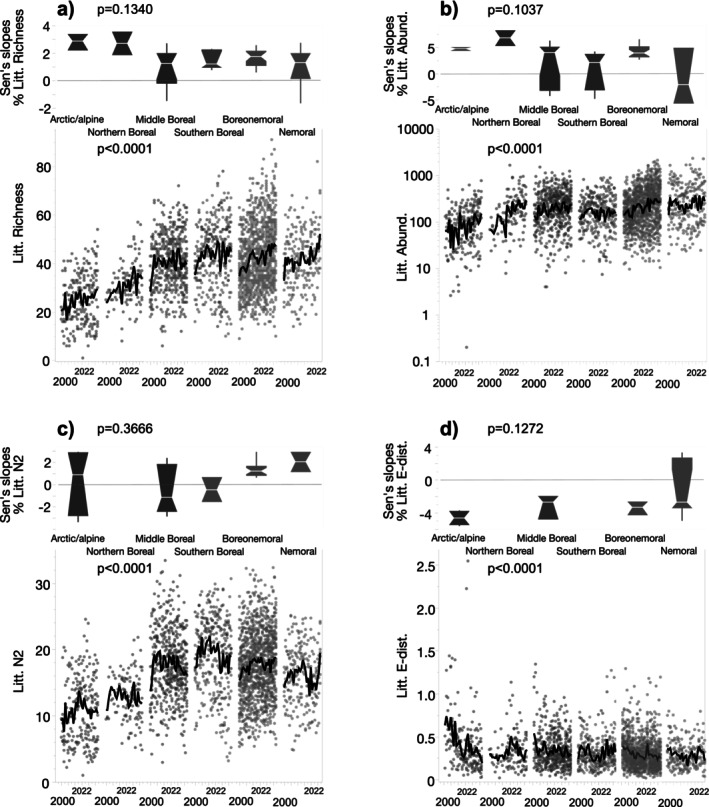
Time‐series plots and Sen's slopes (% per year) of littoral macroinvertebrate (a) richness, (b) abundance (NPUE), (c) Hill's diversity (N^2^), and (d) between‐year Euclidean distance (E‐dist). Green symbols show southern‐LN and blue symbols show northern‐LN lakes. Only Sen's slopes that were significant (*p* < 0.05) are plotted. *p* values show results from a Kruskal–Wallis tests. Black lines show a 4‐year moving average.

For littoral macroinvertebrates, 41% of the lakes (*n* = 45) had significant increasing trends in taxon richness, 35% (*n* = 38) in abundance, and 24% (*n* = 26) in diversity, while E‐dist decreased in 16% (*n* = 18) (Table 2). Only slopes of E‐dist differed significantly between the northern‐LN and southern‐LN lakes. However, analysis of ecoregional differences revealed significant patterns (Figure 4). The most pronounced changes were found in the arctic/alpine and northern boreal ecoregions; for example, taxon richness and abundance had positive slopes of 2.8% ± 0.73%/year (*n* = 5 lakes) and 5.7% ± 1.7%/year (*n* = 4 lakes), respectively (Figure 4a,b). Negative slopes in E‐dist in the arctic/alpine ecoregion (−4.6% ± 0.89%/year) (Figure 4d), combined with greater negative slopes for the northern‐LN lakes in general (see above), show not only marked regional differences in rates of change in taxon richness and diversity but also indicate shifts in species composition/turnover. In the two southernmost boreonemoral and nemoral ecoregions, marked increasing trends in taxon richness and abundance were also observed: 28 lakes (of 31 with significant slopes) had positive slopes for taxon richness (2.0% ± 1.1%/year) and 25 lakes (of 29 with significant trends) had positive slopes for abundance (4.8% ± 2.3%/year).

Nine of the top 10 ranked taxa that discriminated between the northern‐LN and southern‐LN lakes and the six ecoregions overlapped (Table S3a,b). Four of these 10 taxa were chironomid midges (contributing 9.8% to the dissimilarity) and three were mayflies (9.6%). The isopod *Asellus aquaticus* and the mayfly *Leptophlebia vespertina*, ranked first and second, respectively, accounted for c. 8% of the dissimilarities. *Asellus aquaticus* was not found or only found in relatively low abundances in the arctic/alpine and northern boreal ecoregions. The three mayflies *L. vespertina* , *Caenis luctuosa*, and *Caenis horaria* were all more abundant in the southern‐LN lakes.

#### 3.2.3 Profundal Macroinvertebrates

**FIGURE 5:**
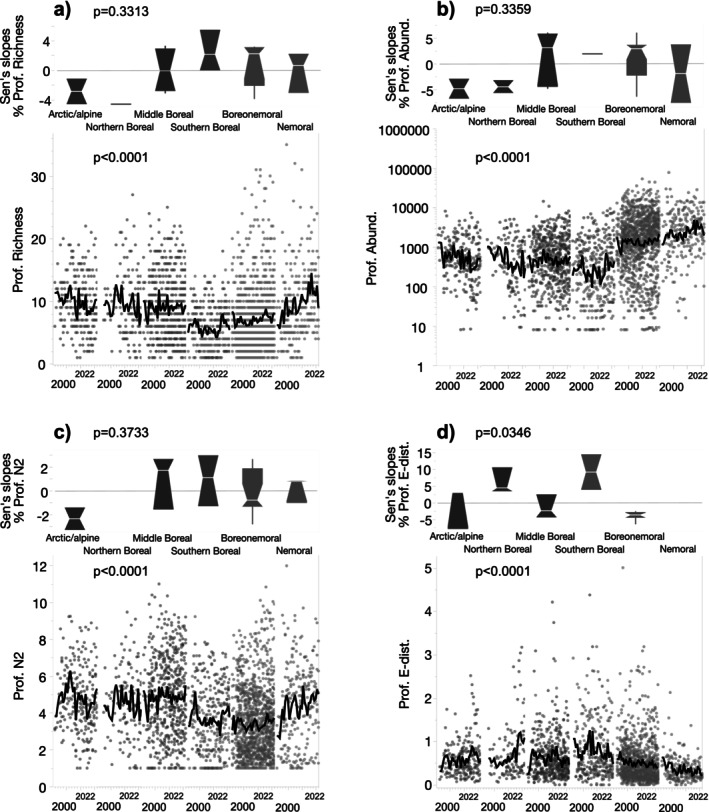
Time‐series plots and Sen's slopes (% per year) of profundal macroinvertebrate (a) richness, (b) abundance (ind./m2), (c) Hill's diversity (N^2^), and (d) between‐year Euclidean distance (E‐dist). Green symbols show southern‐LN and blue symbols show northern‐LN lakes. Only Sen's slopes that were significant (*p* < 0.05) are plotted. *p* values show results from a Kruskal–Wallis tests. Black lines show a four‐year moving average.

For profundal macroinvertebrates, significant trends in all four biological variables were found in 22% of the lakes, but none of the variables differed in relation to the LN ecotone (Table 2). Ecoregional partitioning, however, showed negative slopes for taxon richness (−2.9% ± 2.4%/year), abundance (−4.8% ± 2.7%/year) and diversity (−2.4% + 1.3%/year) in lakes in the arctic/alpine ecoregion (*n* = 2 with significant slopes), while trends for lakes in the southern ecoregions were ambiguous (Figure 5). Slopes for E‐dist differed among the six ecoregions (Figure 5d). Slopes for E‐dist in arctic/alpine lakes averaged −0.57% ± 6.3%/year (*n* = 3 lakes), while slopes in the northern boreal lakes were on average much higher (6.2% ± 3.9%/year, *n* = 3 lakes). By comparison, the 12 lakes in the boreonemoral ecoregion had significant negative slopes for E‐dist (mean −2.9% ± 3.5%/year), including a single lake with a positive slope.

Seven of the top 10 taxa that discriminated between the northern‐LN and southern‐LN lakes and the six ecoregions consisted of chironomid midges (combined these taxa contributed to 31.5% of the dissimilarities) (Table S3a,b). The midge *Chaoborus flavicans* was ranked first, followed by Oligochaeta: these two taxa accounted for c. 24% of the dissimilarities. *C. flavicans* (c. 15% of the dissimilarities) was more abundant in the southern‐LN lakes and absent or found only in relatively low abundances in the three northernmost ecoregions. By contrast, *Pisidium* clams (c. 7% of the dissimilarities) were more abundant in the northern‐LN than southern‐LN lakes. Oligochaeta, accounting for about 9% of the dissimilarities, showed no clear spatial patterns.

### 3.3 Relationships Between Physicochemical and Biological Variables

**FIGURE 6:**
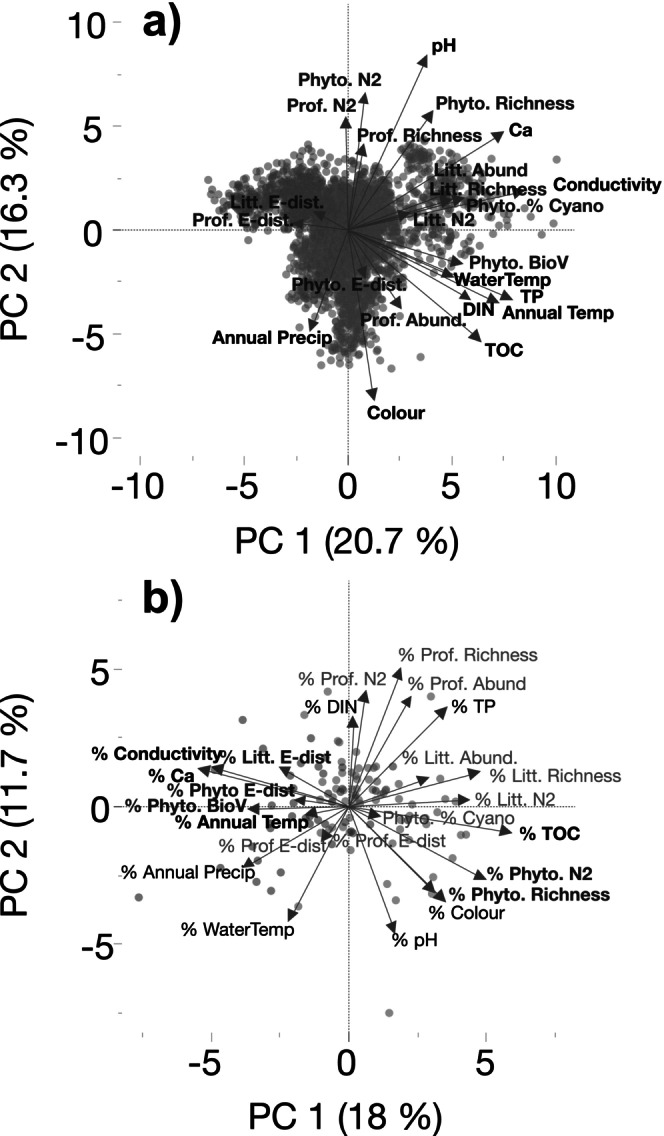
Principal components analysis of (A) 10 abiotic (physicochemical and climate) and 13 biological variables, and (B) the Sen's slopes (%/year), including non‐significant slopes, of these variables. Green symbols show southern‐LN and blue symbols show northern‐LN lakes. Bold text shows significant differences in relation to the Limes Norrlandicus ecotone.

PCA on physicochemical and biological variables showed that the first PC axis (20.7%) was characterised by positive loadings of annual air temperature, conductivity, nutrients, and TOC (Figure 6a). The second PC axis (16.3%) was related to pH (positive loadings) and colour and annual precipitation (negative loadings). Phytoplankton BioV and % cyanobacteria, littoral invertebrate taxon richness and abundance were associated with PC1, while phytoplankton taxon richness and diversity and profundal invertebrate diversity correlated with PC2. Similar to ordinations of magnitudes of change, PCA ordination using rates of change of physicochemical and biological variables showed that the first PC axis (18%) was associated with slopes in TOC and TP (positive loadings) and Ca, conductivity, and annual precipitation (negative loadings) (Figure 6b). The second PC axis (11.7%) was correlated with slopes of pH, surface water temperature, and colour (negative loadings) and DIN (positive loadings). Slopes of phytoplankton and littoral macroinvertebrate taxon richness and diversity were associated (positive loadings) with PC1, while profundal taxon richness and diversity had positive loadings on PC2.

Variation partitioning (pCCA) on all lakes showed that physicochemical variables explained the greatest amount of variability in phytoplankton (56.7%) and littoral (49.1%) and profundal (53.2%) macroinvertebrate assemblages (Table 3). Year alone explained from 9.9% (profundal assemblages) to 22.7% (phytoplankton assemblages) while climate variables alone explained < 7% of the variability. However, the shared variance component between physicochemical and climate variables explained greater variability in both littoral (28.3%) and profundal (31.3%) macroinvertebrate than phytoplankton (14.6%) assemblages. Constrained ordination using forward selection showed that the patterns revealed by pCCA were largely driven by spatiotemporal variability in mean annual air temperature, pH, and TP (Table 4).

**TABLE 3:** Variation partitioning (pCCA) showing the unique variability in phytoplankton and littoral and profundal macroinvertebrate assemblage composition explained by physicochemical factors, year, and climate.

Biological group and fraction	Adj R2	% of Explained	DF	*F*	*p*
A. Phytoplankton					
Group 1 (physicochemical)	0.3288	56.7	9	24.5	0.0001
Group 2 (year)	0.1317	22.7	1	85.6	0.0001
Group 3 (climate)	0.0154	2.6	2	5.9	0.0001
Shared 1 and 2	0.0126	2.2			
Shared 1 and 3	0.0877	14.6			
Shared 2 and 3	0.0080	1.4			
Shared 1, 2, 3	−0.0038	−0.7			
Total explained	0.5976	100	12	32.2	0.0001
All variation	4.985				
B. Littoral macroinvertebrates					
Group 1 (physicochemical)	0.2050	49.1	9	21.4	0.0001
Group 2 (year)	0.0594	14.2	1	54.1	0.0001
Group 3 (climate)	0.0299	7.2	2	14.1	0.0001
Shared 1 and 2	0.0047	1.1			
Shared 1 and 3	0.1182	28.3			
Shared 2 and 3	0.0019	0.5			
Shared 1, 2, 3	−0.0014	−0.3			
Total explained	0.4178	100	12	32.3	0.0001
All variation	3.418				
C. Profundal macroinvertebrates					
Group 1 (physicochemical)	0.3409	53.2	9	20.3	0.0001
Group 2 (year)	0.0637	9.9	1	33.4	0.0001
Group 3 (climate)	0.0335	5.2	2	9.5	0.0001
Shared 1 and 2	0.0004	< 0.1			
Shared 1 and 3	0.2004	31.3			
Shared 2 and 3	0.0032	0.5			
Shared 1, 2, 3	−0.0016	0.5			
Total explained	0.6404	100	12	28.2	0.0001
All variation	5.982				

*Note:* Group 1 = water colour, conductivity, pH, DIN, TP, TOC, and water temperature; Group 2 = year; Group 3 = mean annual air temperature and annual precipitation. pCCA was run using Hellinger transformed biovolume (phytoplankton) and abundance (littoral and profundal macroinvertebrates) data and invoking the down weighting option for rare species.

**TABLE 4:** Constrained ordination (CCA) stopped after the first five significant variables were selected using forward selection of Hellinger transformed data (biovolume for phytoplankton, abundance for macroinvertebrates) of taxon composition and down‐weighting of rare species.

Variable	Contribution %	Pseudo‐F	*p*(adj)
Phytoplankton			
pH	31.7	86.8	0.0011
TP	29.1	82.0	0.0011
Mean annual air temperature	8.42	23.9	0.0011
Water colour	7.43	21.2	0.0011
Conductivity	6.43	18.5	0.0011
Littoral macroinvertebrates			
Mean annual air temperature	36.4	111	0.0011
pH	25.2	78.8	0.0011
TP	11.8	37.5	0.0011
TOC	6.02	19.2	0.0011
Colour	5.69	18.3	0.0011
Profundal macroinvertebrates			
Mean annual air temperature	35.6	101	0.0011
pH	21.2	61.1	0.0011
TP	17.8	52.5	0.0011
TOC	6.4	19.1	0.0011
Conductivity	5.5	16.5	0.0011

*Note:* Variables are listed in order of selection.

**FIGURE 7:**
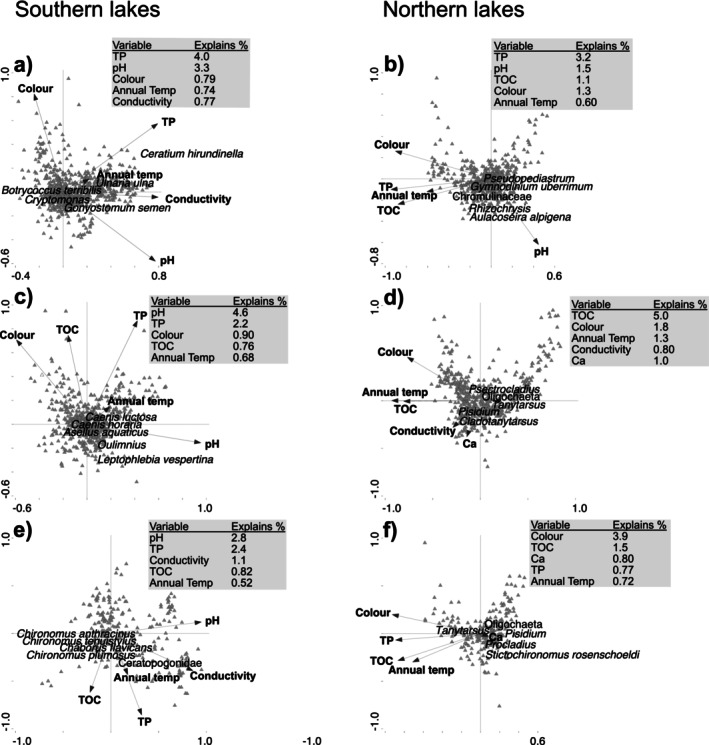
Canonical correspondence analysis of phytoplankton (a, b), littoral macroinvertebrate (c, d) and profundal macroinvertebrate (e, f) assemblages and eight physicochemical variables and two climate variables in southern (a, c, e) and northern (b, d, f) lakes. Grey box shows the order and % of the total variability explained by the first five variables in forward selection. Five indicator taxa, that is, higher relative abundances in northern‐LN or southern‐LN lakes, are shown.

Constrained ordination using forward selection on southern‐LN and northern‐LN lakes revealed that assemblage composition was largely correlated with pH and TP in the southern‐LN lakes (Table S4), these two variables were ranked first or second in the southern‐LN lakes, while benthic macroinvertebrate assemblages in northern‐LN lakes were more strongly correlated with TOC and colour (Figure 7). Of the three groups, littoral taxon richness, diversity, and assemblage composition were more strongly (positively) correlated with water temperature (Table S5) and mean annual air temperature (Figure 7d). Supporting the importance of these environmental drivers, between‐year shifts of phytoplankton (positive) and littoral (negative) assemblages were associated with nutrients in the southern‐LN lakes and with water temperature in the northern‐LN lakes (both negative) (Table S5).

**FIGURE 8:**
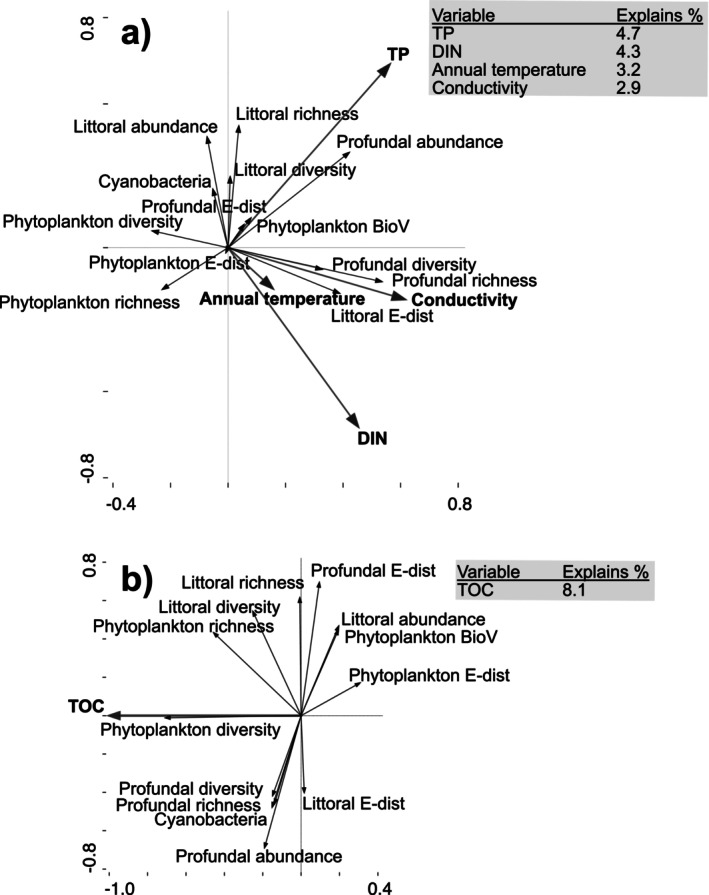
Redundancy analysis of rates of change (%/year) of phytoplankton, littoral macroinvertebrate and profundal macroinvertebrate (including non‐significant slopes) and eight physicochemical variables and two climate variables in southern‐LN (a) and northern‐LN (b) lakes. Grey box shows the order and % of the total variability explained by the first five variables in forward selection.

Redundancy analysis on rates of change of 13 biological variables and on eight physicochemical variables and two climate variables showed that changing nutrients, mean annual air temperature, and conductivity were important predictors in southern‐LN lakes, while temporal changes in TOC were the best predictor for northern‐LN lakes (Figure 8). In southern‐LN lakes, positive slopes of phytoplankton taxon richness and diversity were negatively correlated with nutrients, while positive slopes of littoral taxon richness and profundal abundance were positively correlated with TP (Figure 8a, Table S5). In northern‐LN lakes, only one physicochemical variable (TOC) was selected in the forward selection of RDA. Slopes in TOC were positively correlated with phytoplankton taxon richness and diversity, littoral abundance, and profundal abundance (Figure 8b, Table S5).

## 4 Discussion

Our unique long‐term data set of 110 lakes across whole Sweden showed that the magnitudes and rates of change of physicochemical and biological variables correlate with ongoing changes in regional climate and are strongest in the northernmost regions, thus largely supporting our overarching hypotheses and earlier work on northern lakes (e.g., Huser et al. 2022; Lento et al. 2022; Paltsev et al. 2024). While previous work has summarised spatiotemporal patterns of physicochemical variables in Swedish lakes (Weyhenmeyer et al. 2019; Bergström et al. 2024; Bergström 2010; Huser et al. 2018; Nilsson et al. 2024; Evans et al. 2001; Monteith et al. 2007; Isles et al. 2018), our study has addressed the lake biological responses to these changes. Our study takes this one step further and shows that observed changes in phytoplankton and macroinvertebrate assemblages correlated with changes in the physicochemical environment. As predicted, magnitudes and rates of biological change over time were strongest in the arctic/alpine and the northern boreal ecoregions, characterised by increasing taxon richness and diversity of littoral macroinvertebrates, decreasing taxon richness and diversity of phytoplankton and profundal macroinvertebrates, and greater between‐year shifts in assemblage composition. These findings corroborate earlier work showing that long‐term trends in phytoplankton and zooplankton assemblages were related to climate‐induced changes in lake physicochemical variables (e.g., Paltsev et al. 2024; Bergström et al. 2024). Combined, these findings imply that alterations in lake habitat conditions linked to climate change are shifting the lake biodiversity, especially in the colder regions.

The main environmental drivers behind these trends in physicochemical variables have been related to changes in regional climate affecting catchment geochemistry and vegetation in the northernmost ecoregions, where lakes have been largely unaffected by acidification (Moldan et al. 2013). Conversely, in the southern‐LN lakes, the effects of climate warming are confounded by the recovery from acidification that began in the mid‐1980s (Moldan et al. 2013; Futter et al. 2014). As changes in lake physicochemical variables are governed primarily through catchment alterations such as vegetation development, soil geochemistry, and weathering processes (Adrian et al. 2009), unravelling the ultimate driver(s) of changes in physicochemical variables is challenging. Nonetheless, there is a strong argument to be made for climate change underpinning many spatiotemporal patterns in physicochemical variables, especially in northern‐LN lakes that were largely unaffected by acidification (Moldan et al. 2013).

### 4.1 Phytoplankton

Decreasing nutrient concentrations in northern‐LN lakes, one of the strongest spatiotemporal patterns found here and elsewhere (Huser et al. 2018, 2022), have been attributed to catchment greening and lower external loadings of terrestrial organic matter (Palviainen et al. 2016; Creed et al. 2018). As phytoplankton assemblages are often related to nutrient concentrations, transparency (water colour) and temperature (Stendera and Johnson 2008; Bergström and Karlsson 2019), we anticipated significant correlations with these variables due to first principle relationships. Indeed, variation partitioning and correlations in our data supported the importance of nutrients on phytoplankton assemblages as well as on BioV and % cyanobacteria. Notably, however, slopes of phytoplankton taxon richness and diversity were more strongly correlated with slopes of TOC than with those of TP, especially in northern‐LN lakes (Figure 8b, Table S5), indicating that decreasing TOC concentrations was a relatively more important variable than decreasing TP concentrations. While the mechanisms are not fully understood, low TOC concentrations could result in a higher incidence of harmful UV radiation (Häder et al. 2011), lower nutrient availability for primary production (Bergström and Karlsson 2019), and may alter the food resources of mixotrophic algae (Tittel et al. 2003) that often predominate in many oligotrophic lakes in the north (Deininger et al. 2017).

TOC levels in the northern‐LN lakes of our study were well below the threshold for light limitation for phytoplankton (c. 11 mg/L, Bergström and Karlsson 2019), implying that trends in phytoplankton assemblages are more related to low levels and negative slopes in TOC. Indeed, correlations between rates of change of phytoplankton taxon richness and diversity and those of TOC were stronger in northern‐LN than southern‐LN lakes (Table S5), thus supporting this conjecture. Post hoc analysis of phytoplankton traits as autotrophic, mixotrophic, and heterotrophic (Brettum and Andersen 2004, but classifying all Bacillariophyta as autotrophs) revealed that the biovolumes of all three groups differed between southern‐LN and northern‐LN lakes (all higher in southern‐LN lakes), but only the rates of change of mixotrophs differed among ecoregions, with the highest positive slopes in the arctic/alpine and northern boreal ecoregions (2.1 and 2.3%/year, respectively). The mixotrophic dinophyte *Pseudopedinella*, identified by SIMPER as an indicator of northern‐LN lakes (Table S3b) and an effective bacterivore found in oligotrophic lakes (Gerea et al. 2016), was one of the mixotrophic taxa with increasing populations in these lakes. As mixotrophs generally need more N than P, altered nutrient stoichiometry might partly explain these temporal patterns. A shift to P limitation should favor mixotrophs (Fischer et al. 2017), while N limitation favors N^2^‐fixing cyanobacteria (Jansson 1998; Freeman et al. 2020). Studies addressing N limitation of phytoplankton have shown late summer declines in DIN and TP but not DIN:TP ratios in many of these northern‐LN lakes (Bergström and Karlsson 2019; Bergström et al. 2020). In agreement, we found that 31.8% of the lakes had significant negative slopes in DIN, that is, 22 in northern‐LN lakes and 13 in southern‐LN lakes, although the rates of change did not differ between these two lake populations. Furthermore, post hoc analyses showed that 8 (of 10) northern‐LN lakes had positive slopes in % cyanobacteria (2.7% ± 3.8%/year) lending further support to the significance of climate‐driven alterations of stoichiometry as an important driver of phytoplankton assemblages in these northern‐LN lakes.

Trends in phytoplankton assemblages in the southern‐LN lakes were more ambiguous, likely being partly confounded by the ongoing recovery from acidification (see above). As anticipated, phytoplankton taxon richness and diversity correlated positively with post‐acidification increases in pH, but also negatively with water colour, which could be related to climate (Carvalho et al. 2011). One of the most prominent trends was the relative increase in cyanobacteria in these southern‐LN lakes (% cyanobacteria increased on average 4.7% ± 2.8%/year in 16 of 19 lakes). Cyanobacteria are favoured by warmer temperatures (Davis et al. 2009), but increases were also correlated with Ca, pH, and nutrients (see also Paltsev et al. 2024), which likely indicates responses to both climate warming and post‐acidification changes in physicochemical habitat. Similarly, spatiotemporal distributions of *Gonyostomum semen*, largely restricted to southern‐LN lakes, have been attributed directly or indirectly to climate‐driven changes in spring water temperature, nutrients, TOC, and Fe, but also to post‐acidification increases in pH (Rengefors et al. 2012; Lebret et al. 2018; Hagman et al. 2020; Paltsev et al. 2024). Recently, Paltsev et al. (2024) found that increases in pH along with TOC and Fe correlated with population declines of *Gonyostomum* in many lakes. As both cyanobacteria and *Gonyostomum* are considered poor food quality for consumers (e.g., Ger et al. 2016; Johansson et al. 2016) the population increases and range expansions of these two taxa could affect pelagic food webs and might even affect benthic consumers and the trophic reliance of fish on pelagic versus littoral benthic prey (Lau et al. 2017).

### 4.2 Littoral Macroinvertebrates

For northern‐LN lakes, we anticipated trends in littoral assemblages to be driven more by changes in climate directly. Higher temperatures are expected to facilitate the range expansions and colonizations of warmer water species (Wiens 2016; Lento et al. 2022), but benthic macroinvertebrate assemblages are also indirectly tracking climate‐driven changes in basal resources (e.g., benthic algae and cyanobacteria) (Lau et al. 2014; Lau et al. 2022; Vesterinen et al. 2022). Our findings that mean annual air temperature (Table 4) and TOC and color (Figure 7d,f) were strong predictors of macroinvertebrate assemblage composition in both littoral and profundal habitats, and that littoral macroinvertebrate taxon richness, diversity, and abundance were strongly correlated with water temperature (Table S5), stress the role of climate warming either directly or indirectly as a strong driver of the observed trends. In northern ecosystems, modeling has predicted increases in benthic macroinvertebrate diversity in response to rising temperatures (Domisch et al. 2013). Our finding that macroinvertebrate taxon richness and abundance in northern‐LN lakes were positively correlated with water temperature is to our knowledge the first study to validate this inference. The relatively strong positive slopes in taxon richness might indicate the range expansion of warmer water species (Wiens 2016), while also obscuring the loss of the unique cold‐stenothermic species of the north (Heino et al. 2020).

For southern‐LN lakes, and especially for lakes in the boreonemoral ecoregion that have been affected by acidification (Moldan et al. 2013), we expected trends in littoral macroinvertebrate assemblages to largely reflect post‐acidification re‐colonisation of acid‐sensitive species in response to increases in pH (Carlson et al. 2023) and to a lesser degree climate change. In agreement with our expectations, pH was the single best predictor of littoral macroinvertebrate assemblages in southern‐LN lakes (Figure 7c). In addition, pCCA (Table 4) showed that mean annual air temperature and pH both explained significant variability in littoral assemblages directly, but also indirectly through the shared variance between climate and physicochemical variables. These results support the importance of both climate and post‐acidification recovery as drivers of littoral macroinvertebrate communities. Disentangling climate responses is, however, difficult as signals are often smothered by other pressures (Morris et al. 2022; Belle and Johnson 2024). Due to their acidification history, many southern‐LN lakes are comprised of acid‐tolerant species, which probably are also tolerant to other pressures, such as climate effects (Vinebrooke et al. 2004; Belle et al. 2024). Hence, the cumulative effects of multiple pressures and post‐acidification recovery hysteresis (Johnson and Angeler 2010) make disentangling climate effects from post‐acidification recovery difficult.

### 4.3 Profundal Macroinvertebrates

Northern‐LN lakes are experiencing marked declines in taxon richness, diversity, and abundance of profundal macroinvertebrates, especially in the two northernmost ecoregions. As profundal assemblages in these lakes are comprised of many cold water and oxygen sensitive species (Johnson and Wiederholm 1989), trends could indicate changes in profundal temperatures, oxygen concentrations, and/or basal resources. Notably, and supporting a direct effect of climate, slopes of profundal macroinvertebrate taxon richness and diversity were negatively correlated with slopes of water temperature (Table S5). Our finding of positive correlations between profundal abundance and phytoplankton BioV supports the importance of food resources as a driver in these northern‐LN lakes. Although phytoplankton sedimentation events are an important resource for profundal macroinvertebrates in more eutrophic lakes where diatoms predominate (Goedkoop and Johnson 1996; Johnson and Wiederholm 1992), many of the oligotrophic northern‐LN lakes have phytoplankton assemblages that are predominated by small, motile cryptophytes and chrysophytes that are readily degraded in the open water and that do not settle (Harris 2012). Instead, the primary producers in these lakes should either experience declines in their productivity due to DOC‐driven light limitation in lakes with forested catchments (Karlsson et al. 2009) or shifts toward a larger share of benthic cyanobacteria driven by nitrogen limitation (Diehl et al. 2018), and thus a lower quality basal resource (Brett and Müller‐Navarra 1997), in the clearwater lakes at higher latitudes and/or elevations. Our finding that % cyanobacteria is increasing in many northern‐LN lakes could indicate climate‐driven food web impacts in these nutrient poor lakes. Compared to algae, in particular diatoms, that produce highly unsaturated fatty acids, cyanobacteria are unable to produce these fatty acids and constitute a lower‐quality food resource (e.g., Ahlgren et al. 1990; Goedkoop et al. 2007; Ruess and Müller‐Navarra 2019). These results support the conjecture that warming directly or indirectly, by affecting food resources, contributes to species loss.

In contrast to northern‐LN lakes, profundal macroinvertebrate taxon richness, diversity, and abundance in southern‐LN lakes mainly showed increasing trends and higher variability. Profundal assemblages are usually related to oxygen concentration, food resources, and to a lesser extent temperature (Jyväsjärvi et al. 2013) and our correlations confirm their importance. As many southern‐LN lakes are naturally nutrient‐rich and composed of many warm‐adapted and hypoxia‐tolerant species (Johnson and Wiederholm 1989), we did not expect to find strong (direct) responses to increasing temperatures, simply because profundal habitats in dimictic lakes are less susceptible to warming due to their relatively stable thermal conditions. However, 81% of the lakes with significant positive slopes for TOC were in the south. Lake browning has been attributed to many factors such as acidification, climate change, and land use (Eklöf et al. 2021). Regardless of the driver, increasing TOC concentrations limit not only phytoplankton (Bergström and Karlsson 2019) and whole‐lake production (Karlsson et al. 2009) but also result in more stable and extended periods of stratification, oxygen depletion, and hypoxia (Williamson et al. 2015). TOC concentrations between 5 and 10 mg/L TOC have been shown to result in strong impacts on profundal assemblages (Jane et al. 2024). In our study, southern‐LN lakes had median TOC concentrations of 10 mg/L, indicating that profundal assemblages in half of the lakes are susceptible to increasing TOC. The phantom midge *Chaoborus flavicans* , identified as an indicator for southern‐LN lakes, is known to be hypoxia‐tolerant (Jager and Walz 2002) and therefore often found in high abundances in high TOC lakes (Jane et al. 2024). These findings are in line with work that has shown that local pressures often suppress climate change effects (Morris et al. 2022) and that the pre‐disturbance conditions (i.e., species composition and pressure sensitivities) play a key role in regulating the profundal biological responses to interactions between multiple pressures (Belle et al. 2024). In addition, analyses of subfossil chironomid head capsules have shown that southern‐LN lakes have more hypoxic‐tolerant *Chironomus* species, indicating low profundal oxygen concentrations(Belle et al. 2024). Combined, these findings suggest that profundal assemblages in southern‐LN lakes are responding to increased browning, although more studies are needed to disentangle the effects of increasing hypoxia and alterations in basal resources (e.g., increases in cyanobacteria or *G. semen*).

## 5 Summary

Ongoing climate change is resulting in long‐term ecological transformations in many northern lakes. For example, high latitude lakes are undergoing oligotrophication (declines in TP and DIN) (Isles et al. 2018; Huser et al. 2018), while southern lakes are experiencing brownification due to increases in TOC (Karlsson et al. 2009; Eklöf et al. 2021). These and other climate‐induced environmental changes are expected to impact lake communities. Communities in northern‐LN lakes were responding more rapidly to climate change than southern‐LN lakes, and responses to environmental drivers were habitat‐specific and differed among the three organism groups and between the southern‐LN and northern‐LN lakes. Phytoplankton and littoral macroinvertebrate assemblages showed the strongest correlations to climate, both directly and indirectly, while responses of profundal macroinvertebrate assemblages were more indirect. In northern‐LN lakes, phytoplankton and littoral macroinvertebrate taxon richness correlated directly (water temperature) and indirectly to climate‐driven changes in physicochemical habitat (nutrients and TOC), while profundal macroinvertebrate taxon richness was more related to TOC. In southern‐LN lakes, climate effects were confounded by post‐acidification recovery. For example, phytoplankton taxon richness, diversity, and % cyanobacteria, and littoral macroinvertebrate abundance were positively correlated with pH, while correlations with water temperature were not significant, suggesting no direct effects of warming. Our finding that phytoplankton taxon richness and diversity correlated negatively with water colour (browning) could, however, be an indirect effect of climate warming. This study is the first to compare the magnitudes and rates of change of multiple organism groups and trophic levels to changes in physicochemical habitat and climate. Both phytoplankton and littoral and profundal macroinvertebrate assemblages showed strong trends and therefore should be considered as robust sentinels to quantify climate impacts directly and trophic‐level effects indirectly in these vulnerable ecosystems, which are just at the beginning of major climate‐induced species replacements and changes in the physicochemical habitat for biodiversity.

## Author Contributions

**Richard K. Johnson:** conceptualization, formal analysis, methodology, writing – original draft, writing – review and editing. **Willem Goedkoop:** conceptualization, methodology, writing – review and editing. **Danny C. P. Lau:** conceptualization, methodology, writing – review and editing.

## Conflicts of Interest

The authors declare no conflicts of interest.

## Supporting information


**Table S1.** Selected variables of 110 lakes. Coordinates (latitude, longitude), altitude (m a.s.l.), ecoregion, catchment area (km^2^), lake surface area (km^2^), and present land use.
**Table S2.** Mean (±1SD) of physicochemical and biological variables and % slopes (including non‐significant slopes) for 110 lakes sampled from 1992to 2022. *p* values show results of a Kruskal–Wallis test and letters show differences among the six ecoregions.
**Table S3.** SIMPER results of the 10 top ranked phytoplankton, littoral macroinvertebrate and profundal macroinvertebrate taxa discriminating between (A) southern‐LN and northern‐LN lakes and (B) among the six major ecoregions. Numbers are Hellinger transformed phytoplankton biovolumes and macroinvertebrate abundances.
**Table S4.** Constrained ordination (CCA) of phytoplankton (biovolume) and littoral and profundal macroinvertebrate (abundance) taxon composition for southern‐LN and northern‐LN lakes using Hellinger transformed data and down weighting of rare species. Ordinations were stopped after the first five significant variables were selected using forward selection. Variables are listed in order of selection. Values show percentage of explained variation.
Table S5.

## Data Availability

The data that support the findings of this study are openly available in MVM Soil, Water and Environmental Data database at https://miljodata.slu.se/MVM/. The data that support the findings of this study are openly available at doi.org/10.5878/03t1‐sv66.
