# Supplementary material for: Multi‐Decadal Trends in Northern Lakes Show Contrasting Responses of Phytoplankton and Benthic Macroinvertebrates to Climate Change
Source: Glob Chang Biol. 2025 Jun 9;31(6):e70274. doi: 10.1111/gcb.70274 (PMC12146949; doi:10.1111/gcb.70274)

**Supplementary information**

| Table S1. Selected variables of 110 lakes. Coordinates (latitude, longitude), altitude (m a.s.l.), ecoregion, catchment area (km^2^), lake surface area (km^2^) and perent land use. | | | | | | | | | | | | | | |
| --- | --- | --- | --- | --- | --- | --- | --- | --- | --- | --- | --- | --- | --- | --- |
| Lake | Lat. | Long. | Altitude | Catchment area | Lake Area | % Forest | % Surface water | % Mire | % Forestry | % Open land | % Agriculture | % Alpine | % Urban |  |
| Abiskojaure | 68.31 | 18.65 | 488.1 | 366.9 | 2.65 | 11.5 | 4.4 | 0.3 |  | 4.4 |  | 79.4 |  |  |
| Båtkåjaure | 66.91 | 16.61 | 633.1 | 4.8 | 0.61 | 58.1 | 14.1 |  |  |  |  | 27.8 |  |  |
| Dunnervattnet | 64.28 | 14.69 | 448.8 | 100.8 | 2.75 | 70.6 | 7.5 | 13.7 | 1.1 | 0.0 |  | 7.0 |  |  |
| Latnjajaure | 68.35 | 18.49 | 968.7 | 9.0 | 0.72 |  | 8.3 |  |  |  |  | 91.7 |  |  |
| Njalakjaure | 66.82 | 16.62 | 849.8 | 4.3 | 0.33 |  | 5.6 |  |  |  |  | 94.4 |  |  |
| Östra Helgtjärnen | 63.16 | 13.21 | 648.7 | 2.2 | 0.29 | 52.2 | 13.7 | 33.8 |  |  |  | 0.3 |  |  |
| Övre Fjätsjön | 62.23 | 12.76 | 744.6 | 43.2 | 0.87 | 51.8 | 5.8 | 8.3 |  | 0.3 | 0.1 | 33.7 |  |  |
| Stor-Björsjön | 63.61 | 12.23 | 566.7 | 20.6 | 0.45 | 25.5 | 4.6 | 38.5 |  | 16.6 |  | 14.8 |  |  |
| Stor-Tjulträsket | 65.96 | 16.07 | 538.9 | 277.4 | 5.33 | 16.5 | 5.1 | 4.8 |  | 0.1 | 0.0 | 73.5 |  |  |
| Tronntjärnarna | 62.98 | 13.03 | 1156.3 | 3.1 | 0.03 |  | 1.7 |  |  |  |  | 98.3 |  |  |
| Älgarydssjön | 57.18 | 14.27 | 201.6 | 3.7 | 0.35 | 78.3 | 9.0 | 3.7 | 2.1 | 4.8 | 1.9 |  |  |  |
| Älgsjön | 59.09 | 16.37 | 50.0 | 5.1 | 0.32 | 79.2 | 6.4 | 6.0 | 2.8 | 4.4 | 1.2 |  |  |  |
| Allgjuttern | 57.95 | 16.10 | 131.9 | 1.1 | 0.17 | 84.1 | 14.6 | 1.3 |  |  |  |  |  |  |
| Alsjön | 58.32 | 12.50 | 113.1 | 1.5 | 0.06 | 74.2 | 5.3 | 17.4 | 3.1 |  |  |  |  |  |
| Bästeträsk | 57.92 | 18.94 | 6.2 | 39.9 | 6.65 | 55.9 | 18.6 | 6.7 |  | 9.1 | 9.7 |  |  |  |
| Björken | 58.86 | 17.37 | 32.3 | 7.8 | 1.38 | 77.7 | 17.5 | 0.2 | 4.0 | 0.5 | 0.1 |  |  |  |
| Brunnsjön | 56.60 | 15.73 | 99.4 | 3.9 | 0.11 | 90.9 | 2.7 | 2.6 | 2.9 | 0.9 |  |  |  |  |
| Bysjön | 59.30 | 12.34 | 125.1 | 10.6 | 1.23 | 61.7 | 13.0 | 4.9 | 8.5 | 5.7 | 6.3 |  |  |  |
| Djupa Holmsjön | 59.18 | 17.02 | 61.4 | 1.9 | 0.20 | 82.0 | 10.5 | 7.5 |  |  |  |  |  |  |
| Edasjön | 59.81 | 17.90 | 17.6 | 4.1 | 0.13 | 78.9 | 3.4 | 3.8 | 2.8 | 8.7 | 2.4 |  |  |  |
| Ekholmssjön | 59.87 | 17.04 | 62.5 | 6.9 | 0.57 | 76.0 | 15.1 | 3.5 | 0.7 | 2.2 | 2.6 |  |  |  |
| Fagertärn | 58.76 | 14.70 | 169.1 | 1.8 | 0.20 | 72.9 | 11.1 | 7.9 | 6.9 | 0.0 | 1.1 |  |  |  |
| Fiolen | 57.09 | 14.53 | 226.0 | 5.1 | 1.60 | 37.4 | 31.9 | 2.4 | 3.4 | 7.4 | 17.5 |  |  |  |
| Fjärasjö | 57.61 | 15.25 | 236.4 | 3.3 | 0.35 | 80.0 | 11.4 | 3.0 | 2.2 | 1.6 | 1.7 |  |  |  |
| Fysingen | 59.57 | 17.91 | 2.5 | 165.5 | 4.49 | 36.4 | 6.3 | 1.7 | 0.8 | 13.3 | 30.4 |  | 11.1 |  |
| Glimmingen | 57.94 | 15.57 | 145.4 | 27.6 | 1.75 | 81.1 | 13.8 | 1.3 | 1.5 | 0.6 | 1.8 |  |  |  |
| Grissjön | 58.76 | 15.14 | 139.8 | 1.9 | 0.23 | 80.0 | 11.6 | 6.6 | 1.6 | 0.2 |  |  |  |  |
| Hagasjön | 57.34 | 13.71 | 167.3 | 0.7 | 0.12 | 47.0 | 16.6 | 15.5 | 7.5 | 1.9 | 11.5 |  |  |  |
| Harasjön | 57.01 | 13.58 | 164.3 | 5.4 | 0.56 | 65.0 | 11.5 | 18.5 | 2.0 | 2.9 | 0.1 |  |  |  |
| Hinnasjön | 56.88 | 14.93 | 171.9 | 3.7 | 0.25 | 82.4 | 8.3 | 2.6 | 0.5 | 5.0 | 1.1 |  |  |  |
| Hjärtsjön | 57.05 | 15.26 | 275.2 | 5.7 | 1.28 | 66.5 | 22.6 | 6.3 | 1.1 | 0.6 | 2.9 |  |  |  |
| Hökesjön | 57.64 | 15.76 | 149.9 | 1.5 | 0.55 | 51.4 | 38.2 | 3.4 | 7.1 |  |  |  |  |  |
| Horsan | 57.87 | 18.84 | 5.1 | 3.1 | 0.54 | 57.1 | 17.7 | 1.9 |  | 2.5 | 20.9 |  |  |  |
| Humsjön | 58.62 | 14.48 | 130.4 | 0.6 | 0.25 | 54.6 | 41.1 | 4.3 |  |  |  |  |  |  |
| Lilla Öresjön | 57.56 | 12.34 | 109.4 | 4.5 | 0.61 | 58.6 | 13.9 | 13.1 | 6.8 | 1.9 | 5.7 |  |  |  |
| Lillsjön | 59.10 | 16.81 | 21.7 | 5.8 | 0.34 | 70.4 | 5.8 | 5.6 | 3.6 | 5.3 | 9.3 |  |  |  |
| N. Yngern | 59.17 | 17.41 | 38.7 | 57.9 | 14.29 | 64.0 | 26.8 | 2.2 | 1.1 | 3.5 | 2.4 |  |  |  |
| Öjsjön | 58.17 | 16.21 | 99.3 | 7.9 | 2.01 | 68.3 | 27.2 | 0.6 | 2.7 | 1.0 | 0.2 |  |  |  |
| Örsjön | 56.29 | 14.69 | 88.6 | 0.8 | 0.19 | 75.4 | 21.4 |  | 1.7 | 1.3 | 0.1 |  |  |  |
| Överudssjön | 59.41 | 12.98 | 56.8 | 15.8 | 2.11 | 61.2 | 16.1 | 4.8 | 4.4 | 3.7 | 9.8 |  |  |  |
| Rammsjön | 56.77 | 13.43 | 160.3 | 1.5 | 0.33 | 7.8 | 22.0 | 69.2 | 1.0 |  |  |  |  |  |
| Rotehogstjärnen | 58.82 | 11.61 | 122.5 | 3.5 | 0.15 | 74.2 | 5.0 | 17.4 | 1.5 | 2.0 |  |  |  |  |
| Rundbosjön | 58.81 | 17.37 | 5.5 | 20.3 | 0.91 | 69.1 | 11.4 | 1.2 | 1.9 | 3.7 | 12.9 |  |  |  |
| Sännen | 56.33 | 15.36 | 62.9 | 4.9 | 1.01 | 71.9 | 20.6 | 2.1 | 3.3 | 0.5 | 1.6 |  |  |  |
| Siggeforasjön | 59.98 | 17.16 | 74.2 | 20.4 | 0.72 | 73.9 | 8.0 | 10.9 | 4.2 | 1.8 | 1.2 |  |  |  |
| Skärgölen | 57.78 | 15.58 | 73.0 | 0.9 | 0.17 | 67.2 | 26.4 |  | 6.3 |  |  |  |  |  |
| Skärgölen | 58.76 | 16.23 | 73.0 | 0.9 | 0.17 | 75.9 | 18.2 | 1.6 | 4.2 |  |  |  |  |  |
| St. Lummersjön | 58.11 | 14.11 | 240.3 | 2.4 | 0.05 | 74.6 | 2.9 | 18.9 | 3.7 |  |  |  |  |  |
| Stora Envättern | 59.11 | 17.35 | 64.8 | 1.4 | 0.38 | 57.9 | 26.7 | 15.1 | 0.4 |  |  |  |  |  |
| Stora Gryten | 58.88 | 16.08 | 48.4 | 19.0 | 1.08 | 67.5 | 10.0 | 9.2 | 2.5 | 6.7 | 4.1 |  |  |  |
| Stora Skärsjön | 57.16 | 14.52 | 55.2 | 2.4 | 0.32 | 75.0 | 5.4 | 5.2 | 3.2 | 4.9 | 6.4 |  |  |  |
| Stora Tresticklan | 59.03 | 11.79 | 208.5 | 7.6 | 0.50 | 78.3 | 14.9 | 6.5 | 0.3 |  |  |  |  |  |
| Storasjö | 56.95 | 15.27 | 253.4 | 2.3 | 0.37 | 69.9 | 15.6 | 14.1 | 0.3 |  |  |  |  |  |
| Svartesjön | 56.85 | 13.24 | 157.7 | 0.5 | 0.03 | 42.7 | 5.2 | 48.6 | 3.0 | 0.5 |  |  |  |  |
| Svartsjön | 58.76 | 14.22 | 126.1 | 1.7 | 0.08 | 56.3 | 18.3 | 16.1 | 6.9 | 1.9 | 0.6 |  |  |  |
| Tångerdasjön | 57.46 | 15.06 | 217.5 | 0.8 | 0.17 | 54.3 | 22.2 | 2.6 | 0.2 | 5.8 | 14.8 |  |  |  |
| Tängersjö | 57.47 | 16.03 | 116.1 | 0.5 | 0.11 | 68.9 | 23.0 |  |  | 4.7 | 3.3 |  |  |  |
| Tärnan | 59.56 | 18.37 | 42.3 | 13.2 | 1.05 | 80.0 | 11.5 | 2.0 | 3.8 | 1.2 | 1.5 |  |  |  |
| Tomeshultagölen | 56.74 | 15.41 | 179.3 | 3.8 | 0.08 | 84.6 | 2.0 | 12.5 | 0.3 | 0.1 | 0.6 |  |  |  |
| Västra Solsjön | 59.10 | 12.28 | 150.6 | 9.1 | 1.85 | 69.1 | 23.9 | 1.0 | 3.3 | 1.7 | 1.0 |  |  |  |
| Ymsen | 58.64 | 13.93 | 71.8 | 44.8 | 13.13 | 34.3 | 30.2 | 4.5 | 2.5 | 4.4 | 24.0 |  |  |  |
| Bergträsket | 65.91 | 23.05 | 39.5 | 2.4 | 0.20 | 69.2 | 13.2 | 14.1 | 3.6 |  |  |  |  |  |
| Bjännsjön | 64.24 | 20.44 | 179.9 | 9.1 | 0.47 | 74.7 | 6.0 | 11.4 | 5.8 | 0.9 | 1.3 |  |  |  |
| Brännträsket | 65.53 | 21.42 | 80.9 | 11.1 | 0.91 | 76.7 | 10.7 | 7.1 | 2.0 | 3.0 | 0.5 |  |  |  |
| Degervattnet | 63.87 | 16.23 | 213.7 | 198.6 | 1.66 | 73.0 | 10.8 | 9.4 | 5.3 | 1.1 | 0.4 |  |  |  |
| Fyrsjön | 63.48 | 15.43 | 299.0 | 2406.2 | 0.05 | 59.8 | 6.0 | 25.3 | 3.6 | 1.7 | 1.2 | 2.4 | 0.1 |  |
| Gipsjön | 60.65 | 13.63 | 382.0 | 8.9 | 0.68 | 69.2 | 8.2 | 19.3 | 2.4 | 1.0 |  |  |  |  |
| Hällvattnet | 63.54 | 17.63 | 216.8 | 105.8 | 6.89 | 73.4 | 8.7 | 12.4 | 5.0 | 0.3 | 0.2 |  |  |  |
| Jutsajaure | 67.06 | 19.94 | 421.2 | 19.4 | 1.12 | 70.2 | 6.3 | 21.8 | 0.8 | 0.6 |  | 0.2 |  |  |
| Norra Reivo | 65.77 | 19.10 | 456.6 | 0.0 | 0.81 | 65.2 | 31.1 | 3.7 |  |  |  |  |  |  |
| Ögerträsket | 64.15 | 20.09 | 213.5 | 2.4 | 0.10 | 83.2 | 5.1 | 3.9 | 6.7 | 0.7 | 0.4 |  |  |  |
| Översjön | 59.89 | 13.34 | 220.4 | 2.1 | 0.38 | 69.6 | 18.2 | 0.7 | 9.2 | 2.2 |  |  |  |  |
| Pahajärvi | 66.77 | 23.35 | 248.7 | 6.3 | 1.29 | 68.0 | 19.8 | 12.3 | 0.0 |  |  |  |  |  |
| Remmarsjön | 63.86 | 18.27 | 234.6 | 125.5 | 1.31 | 76.8 | 4.1 | 13.8 | 4.7 | 0.6 | 0.1 |  |  |  |
| Sangen | 61.93 | 14.89 | 444.5 | 11.9 | 1.45 | 66.2 | 12.4 | 16.9 | 4.4 | 0.1 | 0.1 |  |  |  |
| Sidensjön | 63.88 | 19.82 | 134.9 | 1.0 | 0.09 | 92.3 | 7.2 | 0.0 | 0.5 |  |  |  |  |  |
| Stor-Backsjön | 62.68 | 14.51 | 427.6 | 54.5 | 2.07 | 61.8 | 7.5 | 28.7 | 1.5 | 0.5 | 0.0 |  |  |  |
| Svartvattnet | 63.67 | 19.28 | 173.3 | 5.6 | 0.05 | 87.2 | 1.5 | 7.8 | 2.1 | 1.3 | 0.1 |  |  |  |
| Täftesträsket | 64.06 | 20.26 | 140.6 | 18.6 | 2.37 | 61.3 | 14.3 | 11.0 | 2.7 | 4.1 | 6.7 |  |  |  |
| Tväringen | 62.24 | 15.67 | 307.6 | 35.8 | 1.71 | 72.0 | 7.9 | 9.2 | 10.0 | 0.7 | 0.2 |  |  |  |
| Valasjön | 63.00 | 17.52 | 101.8 | 96.7 | 2.01 | 81.3 | 3.8 | 4.3 | 8.2 | 1.7 | 0.7 |  |  |  |
| Vitträsket | 64.69 | 19.70 | 272.0 | 6.4 | 1.96 | 62.5 | 30.6 | 2.1 | 4.5 | 0.3 |  |  |  |  |
| Vuolgamjaure | 65.66 | 18.56 | 435.8 | 34.4 | 2.09 | 71.6 | 6.3 | 17.9 | 0.6 | 0.4 |  | 3.2 |  |  |
| Bäen | 56.25 | 14.38 | 97.3 | 9.7 | 0.54 | 80.6 | 6.8 | 1.8 | 4.0 | 3.2 | 3.5 |  |  |  |
| Fräcksjön | 58.15 | 12.18 | 69.3 | 4.6 | 0.27 | 77.8 | 6.1 | 5.8 | 8.0 | 2.3 |  |  |  |  |
| Granvattnet | 58.23 | 11.77 | 58.1 | 0.9 | 0.17 | 54.0 | 1.6 | 1.3 | 2.2 | 13.1 | 27.7 |  |  |  |
| Havgårdssjön | 55.49 | 13.36 | 51.6 | 2.0 | 0.53 | 13.9 | 26.6 | 0.4 |  | 4.2 | 54.9 |  |  |  |
| Krageholmssjön | 55.50 | 13.76 | 43.5 | 15.5 | 2.06 | 16.4 | 13.2 | 0.0 | 0.3 | 5.9 | 62.4 |  | 1.8 |  |
| Krankesjön | 55.71 | 13.47 | 20.2 | 51.9 | 2.77 | 19.8 | 5.9 | 3.3 | 0.3 | 13.7 | 55.7 |  | 1.4 |  |
| Skärsjön | 57.08 | 12.52 | 48.9 | 13.9 | 3.01 | 60.3 | 26.4 | 3.5 | 2.0 | 2.6 | 5.1 |  |  |  |
| Stora Skärsjön | 56.67 | 13.07 | 55.2 | 2.4 | 0.32 | 68.2 | 16.6 | 12.5 | 2.6 | 0.0 | 0.0 |  |  |  |
| Svinarydsjön | 56.18 | 14.94 | 28.4 | 1.7 | 0.18 | 81.3 | 11.1 | 2.6 | 2.0 | 3.0 |  |  |  |  |
| Gåtejaure | 62.98 | 13.76 | 853.8 | 31.5 | 0.53 | 2.1 | 8.0 | 3.1 |  |  |  | 86.8 |  |  |
| Louvvajaure | 66.39 | 18.17 | 458.2 | 4.2 | 0.80 | 78.4 | 18.9 | 2.6 |  |  |  |  |  |  |
| Stor-Arasjön | 64.60 | 17.60 | 544.0 | 32.8 | 7.29 | 50.4 | 22.4 | 26.3 | 0.4 | 0.5 |  |  |  |  |
| Storvindeln | 65.64 | 17.45 | 344.1 | 2897.6 | 0.00 | 47.0 | 5.2 | 7.6 | 0.6 | 0.6 | 0.1 | 38.8 |  |  |
| Valkeajärvi | 67.60 | 21.79 | 314.9 | 3.4 | 0.66 | 68.8 | 19.3 | 8.1 |  |  |  | 3.8 |  |  |
| Vuolejaure | 62.97 | 13.77 | 855.3 | 6.5 | 0.40 |  | 6.6 | 0.5 |  |  |  | 92.9 |  |  |
| Ämten | 59.63 | 15.43 | 274.9 | 3.0 | 0.47 | 61.6 | 5.2 | 33.2 |  |  |  |  |  |  |
| Dagarn | 59.90 | 15.69 | 135.5 | 34.4 | 1.75 | 73.9 | 14.5 | 2.9 | 6.8 | 1.2 | 0.7 |  |  |  |
| Gosjön | 61.09 | 16.95 | 63.2 | 1.8 | 0.41 | 62.9 | 23.4 | 5.1 | 8.6 | 0.0 |  |  |  |  |
| Hällsjön | 60.16 | 15.73 | 168.7 | 1.5 | 0.21 | 71.4 | 13.6 | 4.6 | 9.7 | 0.8 |  |  |  |  |
| Långsjön | 60.73 | 16.42 | 239.5 | 0.6 | 0.06 | 75.5 | 12.0 | 2.8 | 9.7 |  |  |  |  |  |
| Limmingsjön | 59.59 | 14.52 | 236.4 | 9.6 | 1.14 | 71.7 | 17.7 | 7.9 | 1.6 | 0.9 | 0.2 |  |  |  |
| Mäsen | 60.03 | 15.66 | 106.6 | 3.9 | 0.42 | 76.0 | 10.5 | 3.3 | 6.9 | 2.1 | 0.2 |  | 0.9 |  |
| Övre Skärsjön | 59.84 | 15.55 | 223.3 | 8.7 | 1.80 | 67.3 | 20.7 | 9.5 | 1.0 | 1.2 | 0.2 |  |  |  |
| Spjutsjön | 60.64 | 15.45 | 182.3 | 3.9 | 0.42 | 73.5 | 10.8 | 3.7 | 12.0 |  |  |  |  |  |
| Stensjön | 61.64 | 16.58 | 269.0 | 3.5 | 0.55 | 55.0 | 15.9 | 19.5 | 9.6 | 0.1 |  |  |  |  |
| Ulvsjön | 59.61 | 12.29 | 211.3 | 4.0 | 0.52 | 76.0 | 11.8 | 4.3 | 7.0 | 0.8 |  |  |  |  |
| Rännöbodsjön | 62.33 | 16.99 | 50.4 | 50.3 | 0.45 | 79.3 | 4.0 | 2.8 | 8.8 | 2.1 | 3.0 |  |  |  |

| Table S2. Mean (± 1SD) of physicochemical and biological variables and % slopes (including non-signiciant slopes) for 110 lakes sampled from 1992 - 2022. P values show results from a Kruskal-Wallis test and letter shows differences among the six ecoregions. | | | | | | |
| --- | --- | --- | --- | --- | --- | --- |
|  | **Arctic/alpine** | **Northern Boreal** | **Middle Boreal** | **Southern Boreal** | **Boreonemoral** | **Nemoral** |
| *Physiochemical variables (raw)* |  |  |  |  |  |  |
| Water temperature (<0.0001) | 6.8±1.5 (a) | 7.7±1.2 (ab) | 9.8±0.8 (b) | 11.7±0.7 (c) | 12.5±1.1 (d) | 12.4±2.2 (d) |
| TP (<0.0001) | 4±1.6 (a) | 4.3±1.6 (b) | 10.4±5.8 (bc) | 13.5±15.8 (bc) | 17.3±15.7 (c) | 27.6±25 (c) |
| DIN (<0.0001) | 28.3±18.3 (a) | 22±8.5 (b) | 40.3±15 (bc) | 55±25.7 (c) | 81.8±70.2 (c) | 180.5±139.9 (c) |
| Ca (0.0045) | 2.96±2.1 (a) | 2.3±1.9 (b) | 3.04±2.8 (b) | 2.68±1.2 (b) | 6.92±11.4 (b) | 18.61±22.8 (b) |
| pH (ns) | 6.8±0.3 | 6.8±0.3 | 6.6±0.5 | 6.4±0.5 | 6.5±0.9 | 7.1±1 |
| Conductivity (<0.0001) | 2.5±1.3 (a) | 2.2±1.2 (b) | 2.8±1.4 (c) | 3.2±1.1 (c) | 7.5±8.1 (c) | 16±12.6 (bc) |
| Water colour (0.0001) | 0.038±0.1 (c) | 0.03±0.1 (bc) | 0.144±0.1 (ab) | 0.166±0.2 (a) | 0.16±0.2 (a) | 0.074±0.1 (abc) |
| TOC (<0.0001) | 3.1±2.1 (a) | 3.2±2.2 (ab) | 9±3.4 (b) | 10.3±6.3 (ab) | 11.4±4.3 (c) | 8.9±2.7 (c) |
| Annual Temp (<0.0001) | -0.5±2.05 (e) | 0.3±1.074 (e) | 2.3±1.402 (d) | 4.9±0.663 (c) | 7.1±0.518 (b) | 8.2±0.54 (a) |
| Annual Pecip (0.0021) | 58.7±10.6 (ab) | 49.3±4.5 (b) | 51.8±4.3 (b) | 57.2±3.7 (b) | 55.7±11.5 (b) | 66±13.7 (a) |
|  |  |  |  |  |  |  |
| *Physiochemical variables (%/year)* | |  |  |  |  |  |
| Water temperature (<0.0001) | -0.785±1.09 (b) | -1.187±0.997 (b) | -0.668±0.945 (b) | 0.326±0.261 (a) | 0.408±0.501 (a) | 0.598±0.543 (a) |
| TP (0.0009) | -2.987±1.827 (b) | -1.38±1.307 (a) | -0.376±1.297 (a) | -0.227±1.321 (a) | -0.465±1.148 (a) | -0.16±0.745 (a) |
| DIN (ns) | 0.329±0.678 | -0.069±0.268 | -0.332±0.443 | -0.71±0.406 | -0.631±0.512 | -0.67±0.742 |
| Ca (<0.0001) | 0.668±0.919 (a) | -0.048±0.432 (b) | -0.124±0.434 (b) | -0.552±0.613 (bc) | -0.858±0.572 (c) | -0.932±0.77 (c) |
| pH (0.0195) | 0.009±0.055 (ab) | 0.025±0.03 (ab) | -0.029±0.107 (b) | 0.048±0.063 (ab) | 0.134±0.284 (a) | 0.045±0.187 (ab) |
| Conductivity (<0.0001) | 0.818±0.945 (a) | 1.034±1.669 (ab) | 1.516±1.685 (bc) | 1.749±1.41(d) | 1.306±1.994 (d) | 1.393±1.934 (cd) |
| Water colour (0.0028) | -0.362±0.852 (a) | -0.082±0.132 (ab) | 0.507±0.818 (ab) | 0.89±0.66 (ab) | 0.633±1.182 (bc) | 1.115±1.308 (c) |
| TOC (<0.0001) | -0.356±1.125 (c) | -0.338±0.651 (c) | 0.35±0.713 (b) | 0.981±0.374 (a) | 0.812±0.614 (a) | 0.882±0.501 (a) |
| Annual Temp (0.0002) | 0.724±2.668 (c) | -1.092±5.084 (bc) | 5.863±18.536 (c) | 0.728±0.105 (c) | 0.543±0.062 (b) | 0.507±0.073 (a) |
| Annual Pecip (<0.0001) | 0.037±0.148 (ab) | -0.031±0.088 (ab) | 0.075±0.098 (a) | -0.049±0.095 (b) | -0.175±0.124 (c) | -0.139±0.084 (c) |
|  |  |  |  |  |  |  |
| *Biological variables (raw)* |  |  |  |  |  |  |
| Phyto Nr SPP (0.0456) | 27.5±8.92 (b) | 37.2±9.36 (ab) | 40.2±9.47 (a) | 40.8±11.55 (a) | 39.6±13.86 (a) | 43±11.85 (a) |
| Phyto BioV (<0.0001) | 0.1±0.04 (c) | 0.19±0.12 (bc) | 0.63±0.7 (c) | 1.01±1.05 (bc) | 2.02±2.52 (ab) | 3.65±5.03 (a) |
| Phyto N2 (ns) | 14.2±4.7 | 18.1±3.7 | 18.9±5.9 | 19.1±7.3 | 16.7±7.9 | 18.5±8.9 |
| Phyto E-dist (ns) | 0.357±0.114 | 0.337±0.052 | 0.342±0.069 | 0.351±0.099 | 0.368±0.098 | 0.391±0.114 |
| Phyto % Cyanobacteria (0.001) | 1.6±1.3 © | 2.9±2.5 (bc) | 5.6±5.7 © | 4.7±2.8 (bc) | 10.8±12.3 (ab) | 15.0±14.6 (a) |
| Litt Nr SPP (0.0002) | 24.6±8.5 (b) | 31.7±3.5 (b) | 40.7±7.7 (a) | 42.8±10.4 (a) | 42.4±9.6 (a) | 42±7.5 (a) |
| Litt Abund (0.0042) | 128±58 (c) | 256±125 (abc) | 281±126 (ab) | 204±100 (bc) | 315±167 (a) | 406±352 (a) |
| Litt N2 (<0.0001) | 10.5±4.2 (c) | 12.7±2 (bc) | 17.7±3.5 (a) | 18.9±4.6 (a) | 17.8±3.8 (a) | 16.1±2.9 (ab) |
| Litt E-dist (ns) | 0.391±0.153 | 0.352±0.148 | 0.339±0.085 | 0.319±0.072 | 0.298±0.066 | 0.288±0.056 |
| Prof Nr SPP (0.0181) | 9.7±2.8 (a) | 9.8±4.4 (a) | 9.1±4.5 (a) | 5.8±2.7 (a) | 7.2±4.1 (ab) | 9.6±5 (a) |
| Prof Abund (0.0009) | 995±953 (c) | 997±799 (bc) | 1114±1151 (c) | 1017±1304 (c) | 3424±3573 (ab) | 4548±4838 (a) |
| Prof N2 (0.0079) | 4.8±1.1 (ab) | 4.6±1.5 (abc) | 4.9±1.9 (a) | 3.7±1.5 (bc) | 3.5±1.6 (c) | 4.4±2.1 (abc) |
| Prof E-dist (0.0017) | 0.611±0.222 (abc) | 0.714±0.395 (ab) | 0.641±0.303 (ab) | 0.801±0.374 (a) | 0.532±0.355 (bc) | 0.364±0.156 (c) |
|  |  |  |  |  |  |  |
| *Biological variables (%/year)* |  |  |  |  |  |  |
| Phyto Nr SPP (ns) | -0.601±0.009 | -0.897±0.025 | -0.499±-0.029 | -0.113±0.048 | -0.126±0.134 | 0.04±0.045 |
| Phyto BioV (0.0303) | 0.997±-0.356 (a) | 1.008±-0.338 (a) | 0.354±0.35 (a) | 0.371±0.981 (ab) | -0.929±0.812 (b) | -0.163±0.882 (ab) |
| Phyto N2 (0.0018) | -1.404±-2.987 (c) | -1.376±-1.38 (c) | -0.263±-0.376 (bc) | -0.223±-0.227 (abc) | 0.669±-0.465 (a) | 0.922±-0.16 (ab) |
| Phyto E-dist (ns) | 1.726±-0.785 | 1.331±-1.187 | 1.369±-0.668 | -0.049±0.326 | 0.448±0.408 | 1.35±0.598 |
| Phyto % Cyanobacteria (ns) | -2.20±8.15 (ab) | -5.00±7.38 (b) | 1.18±2.67 (a) | 1.42±2.38 (a) | 0.57±5.39 (a) | 1.27±2.44 (a) |
| Litt Nr SPP (ns) | 0.436±1.265 | 1.43±0.967 | 0.183±1.114 | 0.703±0.62 | 1.098±1.217 | 0.763±0.698 |
| Litt Abund (0.0176) | 1.553±1.111 (ab) | 3.794±0.697 (a) | 0.223±1.777 (b) | 0.556±1.92 (b) | 2.655±2.386 (a) | 0.081±1.92 (b) |
| Litt N2 (ns) | -0.254±1.563 | 0.176±1.148 | -0.157±1.599 | -0.051±2.17 | 0.428±2.009 | 0.454±1.267 |
| Litt E-dist (ns) | -1.896±2.319 | -1.596±2.761 | -1.272±1.39 | 0.183±1.307 | -1.595±1.705 | -1.065±1.966 |
| Prof Nr SPP (0.0238) | -1.311±1.983 (bc) | -1.849±1.261 (c) | 0.165±1.233 (a) | 0.488±0.975 (a) | -0.144±1.391 (a) | 0.193±1.264 (ab) |
| Prof Abund (0.0327) | -1.765±2.372 (a) | -2.443±3.107 (a) | 0.664±3.24 (a) | 0.217±2.377 (ab) | 0.633±3.237 (a) | -0.668±3.005 (ab) |
| Prof N2 (ns) | -0.369±1.979 | -1.307±1.104 | -0.135±1.149 | 0.329±0.871 | -0.285±1.201 | -0.157±1.172 |
| Prof E-dist (0.0084) | 0.298±2.864 (ab) | 3.796±1.119 (a) | -1.242±1.728 (b) | 0.468±2.089 (ab) | -1.436±2.429 (b) | -1.016±2.895 (b) |

| Table S3. SIMPER results of the 10 top ranked phytoplankton, littoral macroinvertebrate and profundal macroinvertebrate taxa discriminating between (A) southern-LN and northern-LN lakes and (B) among the six major ecoregions. Numbers are Hellinger transformed phytoplankton biovolumes and macroinvertebrate abundances. | | | | | | | |  | |  | |  | |
| --- | --- | --- | --- | --- | --- | --- | --- | --- | --- | --- | --- | --- | --- |
| **A. Between southern-LN and northern-LN lakes** | **Av. dissim** | **Contrib. %** | **Cumulative %** | **Northern-LN** | **Southern-LN** |  |  | |  | |  | |  |
| **Phytplankton** |  |  |  |  |  |  |  | |  | |  | |  |
| *Gonyostomum semen* | 3.8 | 5.1 | 5.1 | 0.09 | 0.24 |  |  | |  | |  | |  |
| *Pseudopediastrum* | 2.1 | 2.8 | 7.9 | 0.25 | 0.13 |  |  | |  | |  | |  |
| *Gymnodinium uberrimum* | 1.7 | 2.2 | 10.1 | 0.12 | 0.09 |  |  | |  | |  | |  |
| *Rhizochrysis* | 1.6 | 2.1 | 12.1 | 0.12 | 0.08 |  |  | |  | |  | |  |
| Chromulinaceae | 1.6 | 2.1 | 14.2 | 0.13 | 0.09 |  |  | |  | |  | |  |
| *Aulacoseira alpigena* | 1.5 | 2.0 | 16.1 | 0.12 | 0.04 |  |  | |  | |  | |  |
| *Ulnaria ulna* | 1.4 | 1.9 | 18.0 | 0.08 | 0.08 |  |  | |  | |  | |  |
| *Cryptomonas* | 1.4 | 1.9 | 19.9 | 0.17 | 0.17 |  |  | |  | |  | |  |
| *Ceratium hirundinella* | 1.4 | 1.8 | 21.7 | 0.06 | 0.09 |  |  | |  | |  | |  |
| *Gymnodinium* | 1.4 | 1.8 | 23.5 | 0.14 | 0.09 |  |  | |  | |  | |  |
| **Littoral macroinvertebrates** |  |  |  |  |  |  |  | |  | |  | |  |
| *Asellus aquaticus* | 3.0 | 4.3 | 4.3 | 0.17 | 0.32 |  |  | |  | |  | |  |
| *Leptophlebia vespertina* | 2.5 | 3.6 | 7.9 | 0.18 | 0.25 |  |  | |  | |  | |  |
| *Caenis luctuosa* | 2.3 | 3.3 | 11.2 | 0.01 | 0.20 |  |  | |  | |  | |  |
| Oligochaeta | 2.2 | 3.2 | 14.4 | 0.35 | 0.33 |  |  | |  | |  | |  |
| *Psectrocladius* | 2.1 | 3.0 | 17.4 | 0.23 | 0.13 |  |  | |  | |  | |  |
| *Caenis horaria* | 1.9 | 2.7 | 20.0 | 0.11 | 0.15 |  |  | |  | |  | |  |
| *Tanytarsus* | 1.8 | 2.6 | 22.6 | 0.18 | 0.16 |  |  | |  | |  | |  |
| *Cladotanytarsus* | 1.6 | 2.3 | 24.9 | 0.11 | 0.10 |  |  | |  | |  | |  |
| *Pisidium* | 1.5 | 2.1 | 27.0 | 0.14 | 0.12 |  |  | |  | |  | |  |
| *Conchapelopia* | 1.3 | 1.9 | 28.9 | 0.12 | 0.07 |  |  | |  | |  | |  |
| **Profundal macroinvertebrates** |  |  |  |  |  |  |  | |  | |  | |  |
| *Chaoborus flavicans* | 12.6 | 15.7 | 15.7 | 0.13 | 0.51 |  |  | |  | |  | |  |
| Oligochaeta | 7.2 | 9.0 | 24.6 | 0.27 | 0.26 |  |  | |  | |  | |  |
| *Pisidium* | 6.2 | 7.7 | 32.3 | 0.25 | 0.07 |  |  | |  | |  | |  |
| *Procladius* | 5.8 | 7.2 | 39.5 | 0.27 | 0.18 |  |  | |  | |  | |  |
| *Tanytarsus* | 4.5 | 5.5 | 45.0 | 0.18 | 0.07 |  |  | |  | |  | |  |
| *Stictochironomus rosenschoeldi* | 4.3 | 5.3 | 50.3 | 0.17 | 0.02 |  |  | |  | |  | |  |
| *Zalutschia zalutschicola* | 3.5 | 4.3 | 54.7 | 0.13 | 0.04 |  |  | |  | |  | |  |
| *Sergentia coracina* | 3.5 | 4.3 | 59.0 | 0.08 | 0.08 |  |  | |  | |  | |  |
| *Chironomus tenuistylus* | 2.0 | 2.4 | 61.4 | 0.02 | 0.06 |  |  | |  | |  | |  |
| *Chironomus anthracinus* | 1.9 | 2.3 | 63.8 | 0.03 | 0.05 |  |  | |  | |  | |  |
|  |  |  |  |  |  |  |  | |  | |  | |  |
| **B. Among ecoregions** | **Av. dissim** | **Contrib. %** | **Cumulative %** | **Arctic/alpine** | **Northern Boreal** | **Middle Boreal** | **Southern Boreal** | | **Boreonemoral** | | **Nemoral** | |  |
| **Phytplankton** |  |  |  |  |  |  |  | |  | |  | |  |
| *Gonyostomum semen* | 3.7 | 4.9 | 4.9 | 0.00 | 0.00 | 0.10 | 0.13 | | 0.29 | | 0.19 | |  |
| *Pseudopedinella* | 2.0 | 2.6 | 7.5 | 0.38 | 0.28 | 0.21 | 0.17 | | 0.13 | | 0.11 | |  |
| *Gymnodinium uberrimum* | 1.7 | 2.2 | 9.7 | 0.11 | 0.13 | 0.14 | 0.14 | | 0.08 | | 0.05 | |  |
| *Rhodomonas lacustris* | 1.5 | 2.0 | 11.7 | 0.16 | 0.10 | 0.10 | 0.08 | | 0.08 | | 0.09 | |  |
| Chromulinaceae | 1.5 | 2.0 | 13.7 | 0.20 | 0.11 | 0.12 | 0.10 | | 0.08 | | 0.08 | |  |
| *Uroglena* | 1.5 | 1.9 | 15.7 | 0.07 | 0.13 | 0.08 | 0.09 | | 0.07 | | 0.09 | |  |
| *Cryptomonas* | 1.4 | 1.9 | 17.6 | 0.17 | 0.17 | 0.18 | 0.16 | | 0.16 | | 0.21 | |  |
| *Ceratium hirundinella* | 1.4 | 1.9 | 19.5 | 0.03 | 0.05 | 0.07 | 0.07 | | 0.08 | | 0.15 | |  |
| *Aulacoseira alpigena* | 1.4 | 1.8 | 21.3 | 0.04 | 0.13 | 0.13 | 0.12 | | 0.04 | | 0.01 | |  |
| *Parvodinium inconspicuum* | 1.3 | 1.8 | 23.0 | 0.05 | 0.03 | 0.11 | 0.10 | | 0.07 | | 0.04 | |  |
| **Littoral macroinvertebrates** |  |  |  |  |  |  |  | |  | |  | |  |
| *Asellus aquaticus* | 2.9 | 4.2 | 4.2 | 0.00 | 0.07 | 0.23 | 0.40 | | 0.32 | | 0.25 | |  |
| *Leptophlebia vespertina* | 2.5 | 3.6 | 7.8 | 0.04 | 0.05 | 0.25 | 0.24 | | 0.27 | | 0.17 | |  |
| Oligochaeta | 2.2 | 3.3 | 11.1 | 0.35 | 0.39 | 0.35 | 0.31 | | 0.33 | | 0.33 | |  |
| *Caenis luctuosa* | 2.2 | 3.2 | 14.3 | 0.00 | 0.00 | 0.01 | 0.12 | | 0.19 | | 0.31 | |  |
| *Psectrocladius* | 1.9 | 2.8 | 17.1 | 0.21 | 0.25 | 0.24 | 0.17 | | 0.14 | | 0.05 | |  |
| *Caenis horaria* | 1.9 | 2.7 | 19.8 | 0.03 | 0.04 | 0.14 | 0.16 | | 0.14 | | 0.20 | |  |
| *Tanytarsus* | 1.8 | 2.6 | 22.5 | 0.22 | 0.24 | 0.16 | 0.18 | | 0.16 | | 0.13 | |  |
| *Cladotanytarsus* | 1.6 | 2.3 | 24.8 | 0.09 | 0.02 | 0.12 | 0.11 | | 0.10 | | 0.13 | |  |
| *Pisidium* | 1.5 | 2.2 | 27.0 | 0.09 | 0.07 | 0.16 | 0.13 | | 0.10 | | 0.17 | |  |
| Ceratopogonidae | 1.3 | 1.9 | 28.9 | 0.09 | 0.06 | 0.20 | 0.13 | | 0.14 | | 0.13 | |  |
| **Profundal macroinvertebrates** |  |  |  |  |  |  |  | |  | |  | |  |
| *Chaoborus flavicans* | 11.8 | 15.2 | 15.2 | 0.03 | 0.00 | 0.13 | 0.37 | | 0.55 | | 0.45 | |  |
| Oligochaeta | 7.4 | 9.5 | 24.7 | 0.34 | 0.31 | 0.26 | 0.23 | | 0.25 | | 0.30 | |  |
| *Procladius* | 5.7 | 7.4 | 32.0 | 0.23 | 0.25 | 0.29 | 0.18 | | 0.17 | | 0.28 | |  |
| *Pisidium* | 5.6 | 7.2 | 39.2 | 0.37 | 0.24 | 0.22 | 0.16 | | 0.05 | | 0.10 | |  |
| *Tanytarsus* | 4.2 | 5.3 | 44.5 | 0.11 | 0.18 | 0.19 | 0.11 | | 0.06 | | 0.12 | |  |
| *Stictochironomus rosenschoeldi* | 3.8 | 4.8 | 49.4 | 0.20 | 0.33 | 0.14 | 0.08 | | 0.02 | | 0.00 | |  |
| *Sergentia coracina* | 3.8 | 4.8 | 54.2 | 0.05 | 0.07 | 0.10 | 0.21 | | 0.06 | | 0.01 | |  |
| *Zalutschia zalutschicola* | 3.1 | 3.9 | 58.2 | 0.04 | 0.07 | 0.15 | 0.12 | | 0.05 | | 0.00 | |  |
| *Chironomus tenuistylus* | 1.9 | 2.5 | 60.6 | 0.00 | 0.00 | 0.03 | 0.02 | | 0.07 | | 0.02 | |  |
| Ceratopogonidae | 1.9 | 2.4 | 63.0 | 0.01 | 0.00 | 0.03 | 0.02 | | 0.07 | | 0.12 | |  |

| Table S4. Constrained ordination (CCA) of phytoplankton (biovolume) and littoral and profundal macroinvertebrate (abundance) taxon composition for southern-LN and northern-LN lakes using Hellinger transformed data and down weighting of rare species. Ordinations were stopped after the first five significant variables were selected using forward selection. Variables are listed in order of selection. Values show percentage of explained variation. | | | | | | | |
| --- | --- | --- | --- | --- | --- | --- | --- |
| Variable | Contribution % | pseudo-F | P(adj) | Variable | Contribution % | pseudo-F | P(adj) |
| **Phytoplankton Southern-LN** | |  |  | **Phytoplankton Northern-LN** | |  |  |
| TP | 35.24 | 75.3 | 0.0011 | TP | 30.9 | 34.4 | 0.0011 |
| pH | 28.68 | 63.4 | 0.0011 | pH | 14.3 | 16.2 | 0.0011 |
| Colour | 6.98 | 15.6 | 0.0011 | TOC | 10.8 | 12.4 | 0.0011 |
| Annual air temperature | 6.52 | 14.6 | 0.0011 | Colour | 12.2 | 14.1 | 0.0011 |
| Conductivity | 6.81 | 15.4 | 0.0011 | Annual air temperature | 5.4 | 6.2 | 0.0011 |
|  |  |  |  |  |  |  |  |
| **Littoral macroinvertebrates Southern-LN** | |  |  | **Littoral macroinvertebrates Northern-LN** | |  |  |
| pH | 40.68 | 83.7 | 0.0011 | TOC | 40 | 51.6 | 0.0011 |
| TP | 18.84 | 39.6 | 0.0011 | Colour | 14.6 | 19.2 | 0.0011 |
| Colour | 7.93 | 16.8 | 0.0011 | Annual air temperature | 10.3 | 13.7 | 0.0011 |
| TOC | 6.7 | 14.3 | 0.0011 | Conductivity | 6.7 | 8.9 | 0.0011 |
| Annual air temperature | 5.93 | 12.8 | 0.0011 | Ca | 8.1 | 11 | 0.0011 |
|  |  |  |  |  |  |  |  |
| **Profundal macroinvertebrates Southern-LN** | | |  | **Profundal macroinvertebrates Northern-LN** | | | |
| pH | 29.35 | 49.4 | 0.0011 | Colour | 40.45 | 39.4 | 0.0011 |
| TP | 25.24 | 43.5 | 0.0011 | TOC | 16.04 | 15.9 | 0.0011 |
| Conductivity | 11.3 | 19.7 | 0.0011 | Ca | 8.38 | 8.3 | 0.0011 |
| TOC | 8.7 | 15.3 | 0.0011 | TP | 8.11 | 8.1 | 0.0011 |
| Annual air temperature | 5.57 | 9.8 | 0.0011 | Annual air temperature | 7.56 | 7.6 | 0.0011 |

Table S5.


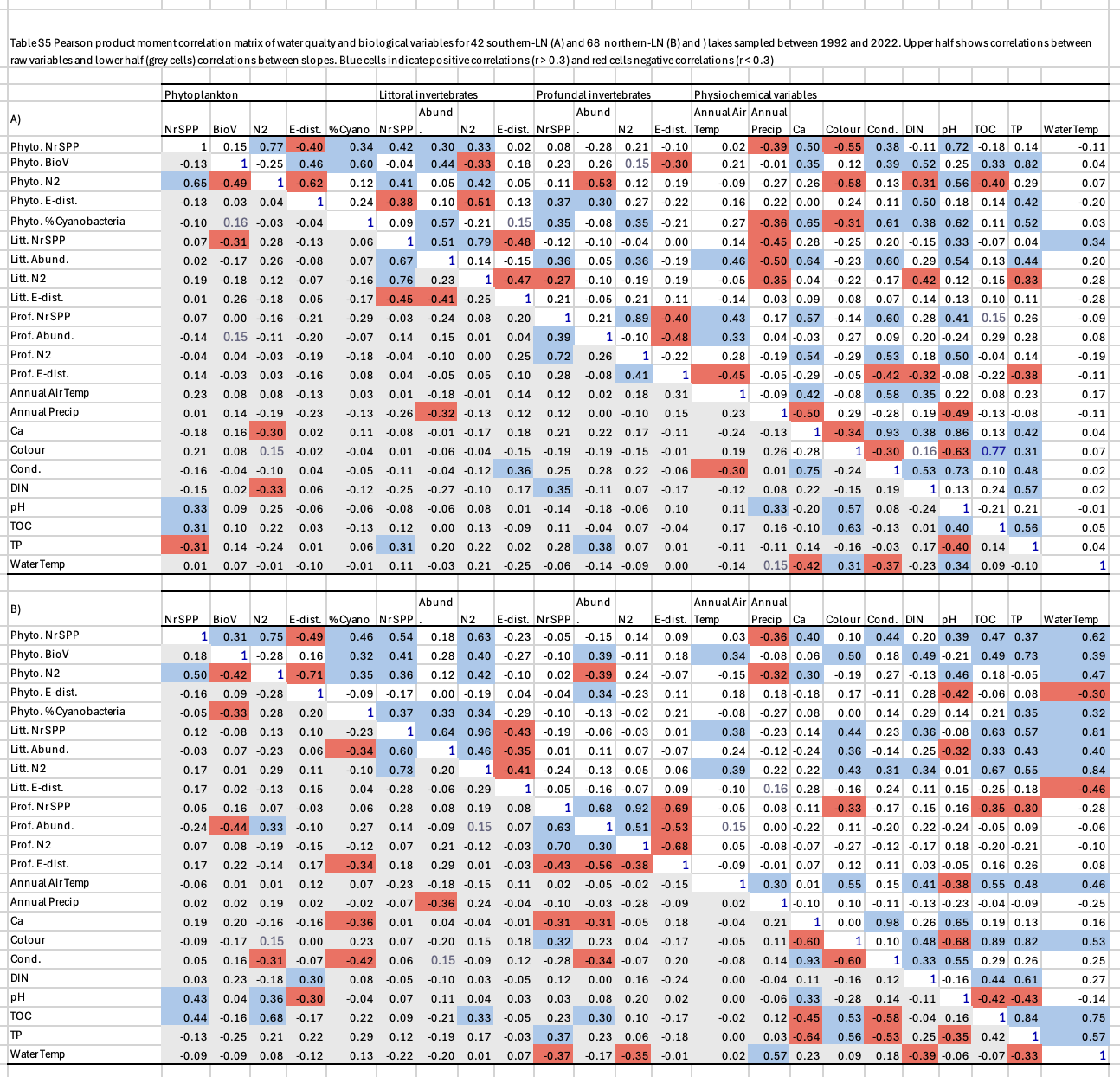

Supplement: Supplementary file 1 — Table S1. Selected variables of 110 lakes. Coordinates (latitude, longitude), altitude (m a.s.l.), ecoregion, catchment area (km2), lake surface area (km2), and present land use. Table S2. Mean (±1SD) of physicochemical and biological variables and % slopes (including non‐significant slopes) for 110 lakes sampled from 1992to 2022. p values show results of a Kruskal–Wallis test and letters show differences among the six ecoregions. Table S3. SIMPER results of the 10 top ranked phytoplankton, littoral macroinvertebrate and profundal macroinvertebrate taxa discriminating between (A) southern‐LN and northern‐LN lakes and (B) among the six major ecoregions. Numbers are Hellinger transformed phytoplankton biovolumes and macroinvertebrate abundances. Table S4. Constrained ordination (CCA) of phytoplankton (biovolume) and littoral and profundal macroinvertebrate (abundance) taxon composition for southern‐LN and northern‐LN lakes using Hellinger transformed data and down weighting of rare species. Ordinations were stopped after the first five significant variables were selected using forward selection. Variables are listed in order of selection. Values show percentage of explained variation. Table S5. [file GCB-31-e70274-s001.docx]
